# Modeling of culture conditions by culture system, glucose and propionic acid and their impact on metabolic profile in IPEC-J2

**DOI:** 10.1371/journal.pone.0307411

**Published:** 2024-07-18

**Authors:** Shirko Marcel Shokr, Stefan Kahlert, Jeannette Kluess, Johannes Hradsky, Sven Dänicke, Hermann-Josef Rothkötter, Constanze Nossol

**Affiliations:** 1 Institute of Anatomy, Medical Faculty, Otto-von-Guericke University, Magdeburg, Germany; 2 Friedrich-Loeffler Institute, Braunschweig, Germany; 3 Institute for Biochemistry and Cell Biology, Medical Faculty, Otto-von-Guericke University, Magdeburg, Germany; CNRS UMR 8576 INSERM U1285 University of Lille, FRANCE

## Abstract

The microbiological environment and their corresponding secreted metabolite spectrum are an essential modulator of the enterocyte function, effecting the whole organism. Intestinal porcine jejunal epithelial cell line (IPEC-J2) is an established in vitro model for differentiation of enterocytes in different cell culture models. An improved oxygen supply seems to be the main reason for differentiation in an air-liquid-interface culture, but this has not yet been conclusively clarified. In this context, the nutrition of the cell and its influence on the metabolism is also of crucial importance. The interest in short-chain fatty acids (SCFAs) has grown steadily in recent years due to their clinical relevance in certain diseases such as multiple sclerosis and other inflammatory diseases, but not much is known of FFAR2 and FFAR3 (free fatty acid receptor 2 and 3) in pigs. We want to address the questions: 1. about the distribution of FFAR2 and FFAR3 *in vivo* and *in vitro* in *sus scrofa* 2. whether there is an influence of propionic acid, glucose content and cultivation on metabolism of enterocytes? The morphological analysis of FFAR2 and FFAR3 *in vivo* was investigated through immunostaining of frozen sections of the porcine gut segments jejunum, ileum and colon. Both receptors are expressed along the gut and were found in the smooth muscle cells of the *tunica muscularis* and *lamina muscularis mucosae*. Furthermore, a high expression of FFAR2 and a low expression of FFAR3 in the enteric nerve system was also observed in jejunum, ileum and colon of *sus scrofa*. In addition, FFAR2 and FFAR3 within the vessels was investigated. FFAR3 showed a strong expression on endothelial cells of veins and lymphatic vessels but was not detectable on arteries. Furthermore, we demonstrate for the first time, FFAR2 and FFAR3 in IPEC-J2 cells on RNA- and protein level, as well as with confocal microscopy. In addition, ENO1 and NDUFA4 were investigated on RNA-level in IPEC-J2 cells as 2 important genes, which play an essential role in metabolism. Here, NDUFA4 is detected in the model animal *sus scrofa* as well as in the porcine cell line IPEC-J2. A potential impact of propionic acid and/or glucose and/or cultivation method on the metabolism of the cells was tested with the Seahorse analyzer. Here, a significant higher ECAR was observed in the SMC than in the OCR. In summary, we were able to show that the cultivation system appears to have a greater influence than the medium composition or nutrition of the cells. However, this can be modulated by incubation time or combination of different SCFAs.

## Introduction

An appropriate cell culture model of the gut for clinical and translational research should display the human *in vivo* situation. The use of the omnivorous pig as a model to mimic the human intestinal barrier function is given by the anatomical and physiological similarities. The abdominal organs like stomach, spleen, bile duct system, small intestine, kidney and bladder found in pigs, share functional and morphological properties with their human counterparts [1]. Furthermore, similar to humans, Furmicutes and Bacteroidetes phyla [2] colonize the gut of pigs. Therefore, the pig model can be used in the area of amino acid metabolism, short chain fatty acids metabolism, total parental nutrition and common bacterial as well as viral infection. Cell culture models use either the isolation of primary cells or permanent cell lines. Primary cells are non-transformed and not tumour derived. In contrast, the use of continuous growing cell lines allows long-time cultivation using straight forward methods at manageable costs. Cultivation of porcine permanent cell lines like IPEC-1 or IPEC-J2 offers both: these cell lines are non-transformed and not tumour derived but can be used for long term studies [3]. Several morphological gut features indicating an adequate gut epithelial cell culture model are: 1. columnar morphology of enterocytes within a monolayer, 2. polarised cell growth, 3. microvilli and 4. expression of tight junctions as well as zonula adherens between the epithelial cells. In particular, IPEC-J2 show morphological and functional similarity to porcine enterocytes [4]. However, proportion of the lateral dimension of the single cell seems to be dependent on culture conditions such as submerged culture (SMC) and Air-liquid interface (ALI) [3]. Modification of ALI culture conditions do not only affect morphological characteristics of the cell line, but has also an impact on the metabolic profile. In airway epithelial cells of mice and bovine cells resulted the application of ALI in a significant increase in oxygen turnover [5, 6]. This seems to be related to HIF-1a, which works as mediator of the metabolic adaption process under hypoxic conditions in SMC [7, 8]. The aim of the study was to analyze the effect of ALI compared to SMC on the metabolic profile of the cell line. Furthermore, we wanted to reflect different diets with the application of high/low glucose and with/without propionic acid. In contrast to other studies, we used a live-cell, real-time analysis of the cellular energy metabolism under these different conditions. This was achieved using the Seahorse analyzer. The seahorse analyzer is also compatible with 3D study model, so we were able to directly analyze our cultured membranes in the device. Regarding the metabolic profile, we examined enolase 1 (ENO1) and NADH Dehydrogenase (Ubiquinone) 1 Alpha Subcomplex, 4 (NDUF4A) as important metabolic key compounds on RNA level. ENO1 mediates the glycolytic pathway and improves the metabolic imbalance of hypoxic cells [9]. In contrast, NDUFA4 is a subunit of the electron transport chain complex belonging to the respiratory chain of mitochondria to produce ATP. Cui and co-workers hypothesize that NDUFA4 but facilitates glycolysis rather than oxidative phosphorylation in colorectal cancer cells [10]. Furthermore, food ingredients themselves or metabolized products by the gut microbiome can also influence the metabolic profile. Here, short chain fatty acids are of great interest due to their health benefits. In porcine colon epithelial cells, butyrate protects cells from hypoxia-induced damage [11]. Therefore, we analysed the effect of propionic acid on the metabolic profile of IPEC-J2 and examined the distribution of FFAR2 and FFAR3 in IPEC-2 but also in the different gut segments.

## Materials and methods

### Animal experiment

Experiment and procedures were conducted according to the European Community regulations concerning the protection of experimental animals and the guidelines of the German Animal Welfare Act and were approved by the ethical committee of the Lower Saxony State Office for Consumer Protection and Food Safety (file number 33.4-42502-04-13/1274). One animal of the control group of a study, which was performed by Bannert and colleagues [12] was used to analyze morphology of FFAR2 and FFAR3 in the porcine gut.

### Cell culture

Intestinal porcine epithelial cells (IPEC-J2) were obtained from Mariana Roseli. Cells were authenticated as porcine cells by Steube et al (ACC 701) [13] and regularly tested for mycoplasma contamination by PCR (Venor GeM Mycoplasma Detection Kit; Minerva Biolabs, Berlin Germany). Cells were seeded in a density of 1*10^5^/cm^2^ on the upper compartment of the transwell system (for western blot/RNA analyses/immunofluorescence staining:12-well, 0.4 μm pore size, Thermoscientific; or seahorse experiments: HTS-96-Transwell, 0.4 μm pore size, Corning; both: polycarbonate). IPEC-J2 cells were maintained at 39°C, 5% CO_2_ and 95% relative humidity in DMEM F12 (1:1) supplemented with 5% fetal bovine serum (FBS), 16 mM 4-(2-hydroxyethyl)-1-piperazineethansulfonic acid (HEPES), 1% insulin-transferrin-selenium (ITS) (all from PAN-Biotech, Aidenbach, Germany), and 5 ng/mL epidermal growth factor (EGF; Biochrome, Berlin, Germany) (complete DMEM). After culture for 10 days in complete DMEM (apical: 1 mL; basolateral: 2 mL), FBS was reduced stepwise every second day (2.5%, 1%, 0%) and simultaneous with 0% FBS, apical media of ALI cultures was aspirated and not refilled. This contrasts with SMC, which were refilled with 1 mL media. SMC and ALI culture were maintained for further 21 days in FBS-free media.

### Cell culture treatment

**Fig 1:**
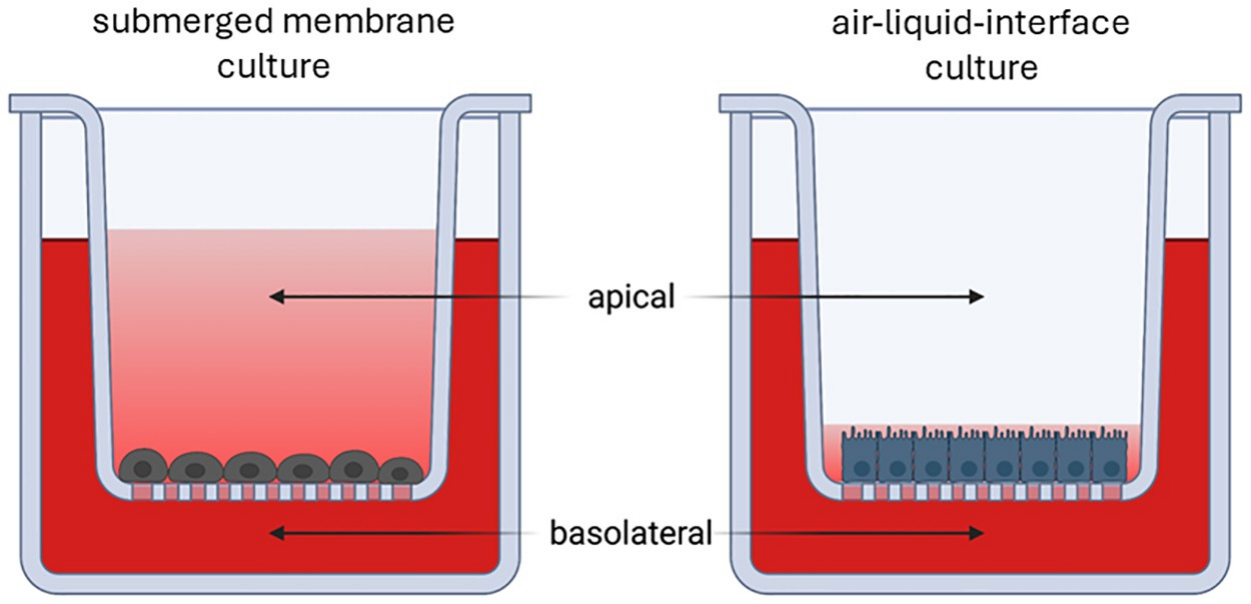
Schematic representation of submerged membrane culture in comparison to Air-liquid interface culture (ALI). Created with Biorender.com.

On the day before ending the experiment, glucose concentration was reduced (LOW: 34.1 mg/L) and/or Na-propionate (apical: 360 μg/mL; Sigma-Aldrich, USA) was applied to the medium (start: day 36) for 24h. Media with high or low glucose was used as control and was added to the apical compartment of SMC and ALI (Fig 1). The cultivation of the cells (SMC/ALI) and the application propionic acid or no propionic acid (PA/wo PA) combined with two different glucose concentrations (HIGH/LOW) resulted in 8 treatment groups: SMC/wo PA/HIGH, SMC/PA/HIGH, SMC/wo PA/LOW, SMC/PA/LOW, ALI/wo PA/HIGH, ALI/PA/HIGH, ALI/wo PA/LOW and ALI/PA/LOW.

### Transepithelial electric resistance (TEER)

The electrical resistance of the monolayer is a quantitative measure of barrier integrity (TEER). Two electrodes were used for electrical measurements using Millicell^®^ ERS (Millipore, Darmstadt, Germany). One electrode was placed in the upper compartment and the other in the lower compartment. Both electrodes were separated by the monolayer. For TEER measurement pre-warmed medium was added to apical compartment of ALI for the duration of measurement only (maximum duration 5 min). In between the measurements the electrode was disinfected using 70% ethanol, washed in ampuwa^®^ and calibrated in pre-warmed FBS-free culture medium. TEER was assessed once a week.

TEER values were calculated as [14]:

$$\text{TEER}\;\left({\Omega \text{cm}}^{2}\right)=\text{Resistance}\;\left(\Omega \right)\;\text{x}\;\text{Area}\;\left({\text{cm}}^{2}\right)$$

Due to the missing compatibility of our electrode and 96-well format, no measurement was possible but an immunolabeling with tight junction markers in a preliminary test was performed. TEER measurements of the 12-well-format was used for analyses.

### RNA isolation and cDNA-synthesis

After 24h of incubation, total RNA was isolated using TRIzol RNA isolation reagent (Invitrogen, Waltham, MA, USA). The supernatant was extracted after centrifuging the cells suspension (12000 x g; 4°C). RNA was precipitated using 250 μL isopropanol alcohol (10 min, 12000 x g, 4°C), purified using 75% ethanol (4°C; 7400 x g; 1 mL) and stored in RNA-free water peqGOLD (Peglab, Erlangen, Germany) at -80°C until further processing. Each 1 μg of template RNA was subjected to reverse transcription with First Aid Reverse Transcription Reagents (Thermo Fisher, USA) essentially as described by the manufacturer with the supplied random hexamer primers in a thermal cycler (Veriti^™^ Applied Biosystems, USA).

### DNA isolation

Cryo sections of the gut (30/gut segment) were used for isolating DNA and lysed in 500 μL lysis buffer (5 Vol.-% 1 M TRIS buffer, 2 Vol.-% 5 M NaCl, 20 Vol.-% 0.5 M EDTA, 10 Vol.-% 10% SDS) with 20 μL proteinase K (Merk Millipore; Germany) overnight at 55°C (350 rpm; thermal block). On the next day, probes were vortexed and centrifuged at 13000 rpm (5 min). 350 μL of the supernatant was mixed with 350 μL isopropanol (Sigma-Aldrich, Germany) to precipitate the DNA. Furthermore, solution was centrifuged (10 000 rpm, 15 min) and supernatants were removed. Pellet was washed with ethanol (70%, Roth, Germany; 10 000 rpm, 5 min), dried on a thermal block and resolved in 20 μL TE buffer (1 Vol.-% 0.5 M EDTA; 5 Vol% 1 M TRIS buffer in Aqua dest.). The concentration was measured with a SmartSpec^™^ photometer (USA).

### PCR and gel electrophoresis

2 ng/μL and 100 ng/μL of the cDNA of IPEC-J2 cells and of the isolated DNA of the gut sections were used for PCR. 1 μL of the sample was mixed with 24 μL of the mastermix (Table 1).

**Table 1:** Master mix of PCR.

content	μL
**water**	18.15
**10x PCR buffer**	2.5
**dNTP Mix (5mM/dNTP)**	0.5
**MgCl_2_ (50 mM)**	0.75
**primer forward (10 pmol/μL)**	1
**primer reverse (10 pmol/μL)**	1
**water**	0.2

Primer pairs were designed using the NCBI sequence of *sus scrofa* and primer3 [15] (https://primer3.org). PCR amplification was performed for FFAR2 and FFAR3 (Table 2) under following conditions on a thermal cycler (Mastercycler, Eppendorf, Germany): 10 min/95°C, followed by 35 cycles of: 60 s/95°C, 60 s/60°C, 90 s/72°C and a final elongation of 10 min/72°C. For gel electrophoresis, 10 μL of each sample was mixed with 3 μL loading buffer (Bromophenol Blue–Xylene Cyanol Dye solution in 30% glycerine). In the next step, 10 μL of the sample/loading buffer mix and a molecular weight ladder was loaded to the gel and run at 100 V.

**Table 2:** Primer sequences.

name	function	sequence 5`-3`	sequence 3`-5`	temperature (°C)	efficiency (%)
**β-actin**	cytoskeletal protein	GAT GAG ATT GGC ATG GCT TT	CAC CTT CAC CGT TCC AGT TT	61	115
**18S**	ribosomal protein	GCA ATT ATT CCC CAT GAA CG	GGC CTC ACT AAA CCA TCC AA	60.5	98
**ENO1**	enolase 1	ACT GCT TCC TTA GAC CTG CT	TGG AAG CGA GGT GAG AAG AA	62.5	138
**NDUFA4**	subunit of complex IV	CCG GGA GCT AAG AAG GGA AT	CTT TGG GCA CAG GTC TCT CT	61.5	141
**FFAR2**	free fatty acid receptor	TCA CGG CCT ACA TCC TCA TC	CTG AGC AGG AGG ATG TGG AT	PCR	
**FFAR3**	free fatty acid receptor	GCA GCG TGG TCT ACA TCA TC	AGA AGA GCC AGC TGA TCC TC	PCR	

### qPCR

The reaction volume of 10 μL contained 9 μL mastermix (Fermentas, Germany) with SensiFAST^™^ SYBR^®^ No-ROX one Step Mix, 10 pmol/μL of the respective primers (0.4 μL each), 0.2 μL Ribosafe RNase inhibitor (Bioline, Germany), 0.1 μL reverse transcriptase (Bioline, Germany), 1.9 μL nuclease free water and 2 μL RNA (2 ng). qPCR amplification was performed for NDUFA4 and ENO1 (Table 2) under following conditions on a qTOWER (Analytik Jena, Germany): 10 min/45°C and 2 min/95°C, followed by 40 cycles of: 5 s/95°C and 20s/NDUFA1 56°C /ENO1 57°C. ß-actin and 18S were used as house keepers. The geometric mean was calculated and subtracted from CT-values. The mean CT-values were used for the plots. Here, low values indicate a high and high CT-values a low amount of the gene. The experiment was carried out at least 5 times.

### Western blot

Treated cells were rinsed twice with PBS, lysed in SDS buffer (1 M TRIS base pH 6.8, 10 Vol.-% glycerol, 2 Vol.-% SDS, 0.005 Vol.-% bromophenol blue, 5 Vol.-% ß-mercaptoethanol), scraped off the membrane and heated at 95°C for 5 min. The Qubit Protein Assay Kit using the Qubit 2.0 Fluorometer (both Invitrogen; Waltham, MA, USA) was performed for quantification of protein content.

15 μg of the protein per sample as well as Page Ruler prestained protein ladder (SM0671; Fermentas Waltham, MA, USA) were electrophoresed on SDS-Page and transferred to a 0.45 μm PVDF membrane by semidry electroblotting using Trans-Blot SD Semi Dry Transfer Cell (Bio-Rad, Munich, Germany). The BM Chemiluminescence Western Blotting Kit (Mouse/Rabbit) was employed for protein detection and was performed after manufacturer’s instructions. For specific protein identifying different antibodies were utilized: rabbit anti-FFAR2 1:1000, mouse anti-FFAR3 1:1000 (both: proteintech, Manchester, UK) and mouse anti-ß-actin 1:40 000 (Sigma-Aldrich, Munich, Germany). The loading control (β-actin) was utilized for normalization, and the band with the highest raw intensity was used to normalize all other β-actin bands (normalization factor). Subsequently, the normalization factors were applied to the corresponding raw intensities of the investigated proteins. The normalized control groups for the corresponding treatment (wo PA or HIGH or SMC) values were used as reference (set to 1) in the boxplot diagrams. The experiment was carried out 3 times.

### Immunolabeling and analysis

For immunostaining, 5 μm cryo sections were cut and air-dried. In the next step, sections were fixed with ethanol/acetone (1:1, 90 sec; Roth, Germany), washed (4x 10 min; TBS/0.1%Tween) and encircled with PAPpen (Dako, Denmark). Primary antibodies (FFAR2, rabbit-anti human/pig; FFAR3, mouse-anti human; both: 1:100, overnight at 4°C, proteintech, UK) were prepared in 1% BSA/TBS/0.1%Tween and centrifuged (5 min; 10 000 rpm) before adding antibody solution to the cryo sections. A control for secondary body and autofluorescence were carried along and no primary antibody but appropriate buffer was added.

On the next day, cryo section were washed 4x with TBS/0.1%Tween before incubating with the secondary antibodies (donkey-anti mouse IgG H&L Alexa 594, abcam, UK; goat anti-rabbit IgG Atto^®^ 647, Sigma-Aldrich, USA; both: 1:400; 1h RT) in TBS/0.1% Tween (with 5 Vol.-% porcine serum). After incubation with secondary antibodies, cryo sections were washed with buffer and nuclei were stained with CyStain^®^ (1:10, UV Ploidy Sysmex Partec, Germany) in TBS/0.1%Tween for 5 min. Afterwards, cryo sections were washed with TBS/0.1% Tween (3x) and with PBS (1x, pH 8.9). VECTASHIELD^®^-DABCO^®^ was used for covering the section with coverslip. IPEC-J2 monolayers were stained after same protocol except for fixation with ethanol/acetone. Membranes were fixed with ethanol because acetone tackles the polycarbonate membrane but acetone causes gaps in the cell membrane, which are important for penetration of antibodies. We inserted a further step and incubated the monolayers with 0.3% Triton in TBS/0.1%Tween for 30 min (RT) before adding the primary antibodies.

The Thunder imager system (DMi8 microscope with DFC9000GTC sCMOS camers; LAS X software; Leica, Germany) was used for analyses of taking pictures. A general view (HC PL APO 40x/0.95 DRY) and pictures in detail (HC PL APO 63x/1.40 OIL objective) were fully automated taken and single pictures were merged (LAS XSoftware, Leica). The exposure time was 1149.87 ms. In the next step, a Large Volume Computational Clearing (LVCC) was performed to reduce unspecific background fluorescence.

3D confocal pictures were recorded with Zeiss LSM 800 equipped with an Airyscan detector in 170 nm steps. Raw data were processed using constant super-resolution filter settings for each channel. After processing slices were reconstructed with ZEN software (ZEN 3.2) and results are presented as orthogonal projections, adjuste4d to comparable lookup table settings.

### Functional analysis of metabolism via seahorse analysis

*Cartridge rehydration*. A mitostress test was performed via Seahorse Analyser (Agilent Technologies. On day before, a sensor cartridge was hydrated by adding 200 μL ampuwa^®^ to each well followed by overnight incubation (39°C; non-CO_2_). At the same time, 20 mL of calibrant (Agilent,USA) was stored overnight in the same incubator. On the day of experiment, ampuwa^®^ was aspirated, calibrant was added (200 μL/well) and sensor plate was incubated for further 45 min (39°C; non-CO_2_).

*Seahorse settings*. Prior the OCR measurement, the following protocol was programmed: 1. three min mixing, 2. two min waiting and 3. three min measuring.

For oxygen consumption rates determination, cells were maintained in XF base medium (Agilent Technologies, USA) supplemented with 17.5 mM glucose, 1 mM pyruvate and 2.5 mM glutamine. The pH was adjusted to 7.40 at 39°C with 0.1 M NaOH and medium was sterile filtered. Stock solutions were produced according to manufactures protocol which were used to prepare operating concentrations (oligomycin: 15 μM; FCCP: 20 μM; rotenone/antimycin A: 5 μM).

*Sample preparation*. Cells were seeded on HTS-96-transwells as described above. On the day of experiment, cells were washed with assay medium (100 μL/compartment) and membranes were cut out with a scalpel or removed with a forceps from the upper compartment. 100 μL of assay medium was provided in each well of culture plate. HTS-96-membranes were transferred into the plate and hydrated capture screens were inserted into each well. In the next step, 400 μL of the assay medium was added to the wells and plate was incubated for 45 min at 39°C/non-CO_2_. During incubation, ports of the sensor cartridge were filled (port A: 56 μL, port B: 62 μL; port: C: 69 μL) with operating concentrations of oligomycin, FCCP and rotenone/Antimycin A.

*Quality criteria*. We have defined quality criteria for evaluation of our seahorse data: 1. there must be no negative readings for oxygen consumption rate (ORC) or extracellular acidification rate (ECAR); 2. there must be no negative respiratory parameters; 3. there must be not any implausible progression curves.

### Statistical analysis

Statistical analyses were performed with GraphPad Prism (version 9.3.1) and SPSS^®^ (version 28.0.1.1). Data were checked for normal distribution (Shapiro-Wilk test) and homogeneity of variance (Levene test). In the case of normal distribution, a 3 factorial (cultivation x glucose content x propionic acid) ANOVA with Tukey-Post Hoc-test or a T-tests was performed. In the case of no normal distribution a Mann-Whitney-U-test were carried out (*p≤0.05; *p≤0.01; **p≤0.001).

## Results

### Distribution of FFAR2 and FFAR3 in the porcine gut

#### Tunica mucosa

**Fig 2:**
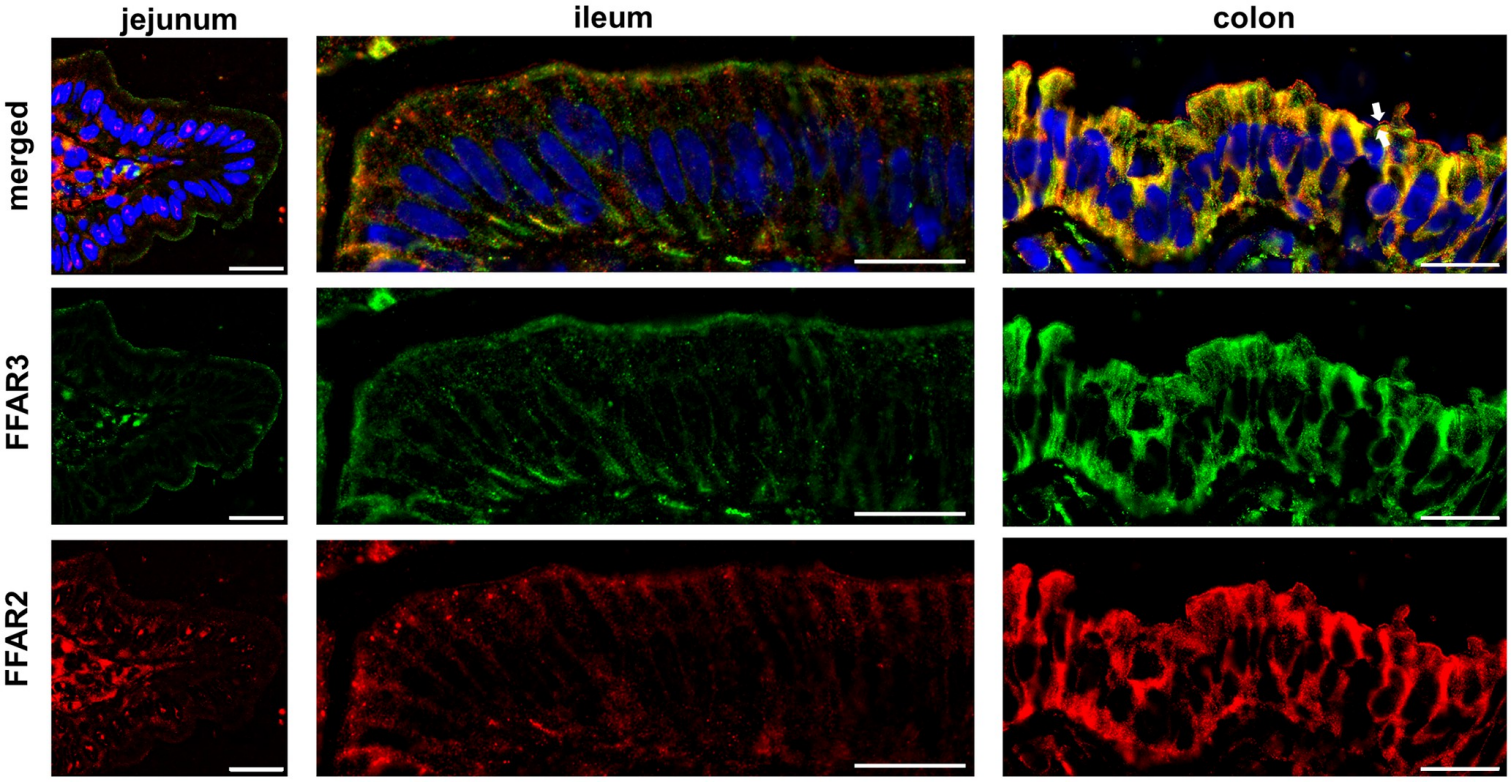
FFAR2 and FFAR 3 in porcine Tunica mucosa. Gut sections (5 μm) of jejunum, ileum and colon were stained with anti-FFAR2 (red) and anti-FFAR3 (green) antibodies (3 replicates). Furthermore, DAPI (blue) was used for nuclei staining. Jejunal enterocytes of the lamina epithelialis mucosae showed a stronger expression of FFAR3 than FFAR2 in the apical membrane of the cells. A positive FFAR2 and FFAR3 staining at both sides of the enterocytes was observed in ileal enterocytes. In addition, colonocytes were highly positive for both receptors, but in the apical membrane they showed a particular expression in form of two separated lines (arrows) [bar = 20μm].

In the lamina epithelialis mucosae, FFAR3 was strongly expressed in the apical membrane and in the cytoplasm of jejunal enterocytes (Fig 2; S1–S11 Figs). A stronger expression of FFAR2 was found at the basolateral side of the enterocytes than apical but also perinuclear. In ileal enterocytes, FFAR2 and FFAR3 was detected at both sides of enterocytes (Fig 2). Contrary to jejunal and ileal enterocytes, colonocytes showed both receptors in the whole membrane but also perinuclear (Fig 2). The apical membrane of colonocytes offered a particular distribution of both receptors in form of two separated lines (Fig 2, arrows) and the expression also decreased from luminal side to the bottom of the crypts of the colon.

#### Tunica muscularis

**Fig 3:**
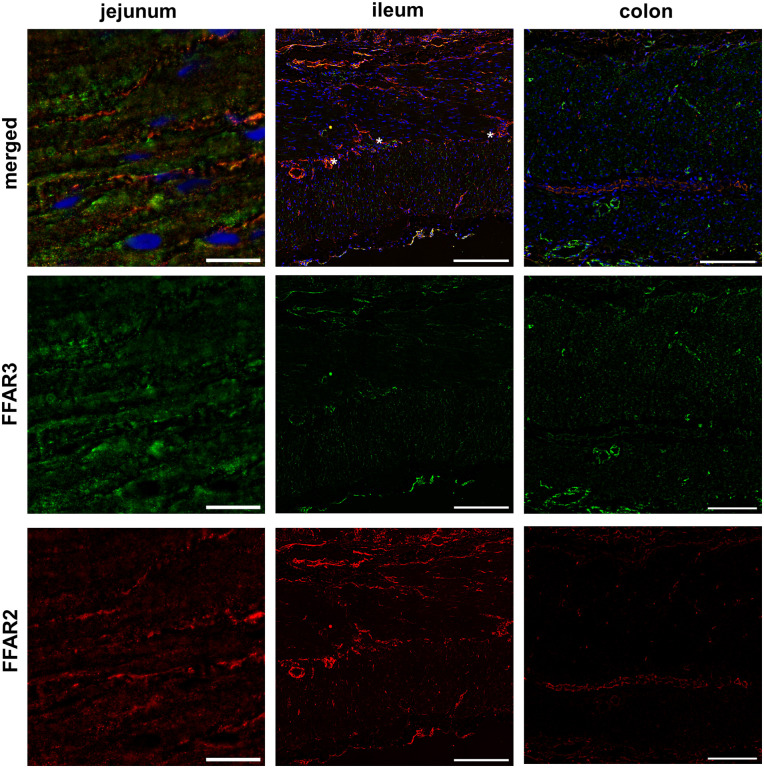
FFAR2 and FFAR3 in porcine Tunica muscularis. Immunofluorescence labelling was performed with 5μm frozen gut section and were stained with an anti-FFAR2 (red) and anti-FFAR3 (green) antibody. Nuclei were marked with DAPI (blue). The tunica muscularis as well as lamina muscularis mucosae of all three gut segments: jejunum [bar = 100μm], ileum [bar = 20 μm] and colon [bar = 100μm] were found to be FFAR3-positive, but no FFAR2 positive smooth muscle cells were detected in the tunica muscularis.

FFAR3-positive smooth muscle cells were detected in the *tunica muscularis* in all three gut segments but no FFAR2 positive smooth muscle cells were found (Fig 3; S1–S11 Figs).

#### Vessels

**Fig 4:**
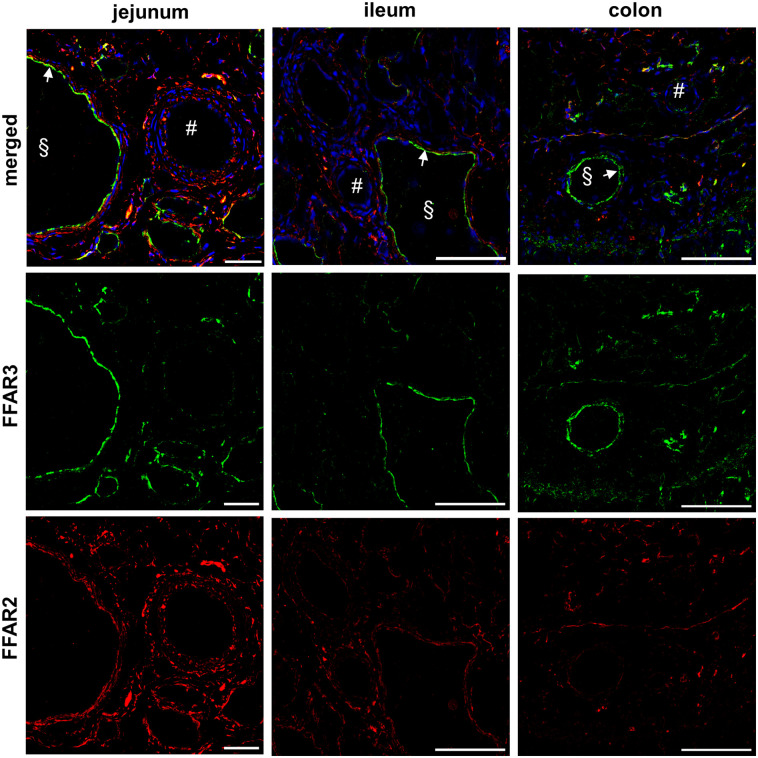
FFAR2 and FFAR3 in vessels. Different frozen gut sections (5 μm) were labelled with an anti-FFAR2 (red) and anti-FFAR3 (green) antibody and nuclei staining was performed with DAPI (blue). Endothelial cells of veins (§) and lymphatic vessels showed a very high expression of FFAR3, but no FFAR2 [jejunum: bar = 200μm; ileum: bar = 100μm; colon: bar = 100μm].

In all three gut segments, endothelial cells of veins (§) and lymphatic vessels showed a very high FFAR3 expression (Fig 4, arrows; S1–S11 Figs). Endothelial cells of arteries (#) were FFAR3 negative. Contrary to FFAR3, FFAR2 was highly expressed in smooth muscle cells of the *tunica media* of veins and a low expression in smooth muscle cells of arteries.

#### Nervous system

**Fig 5:**
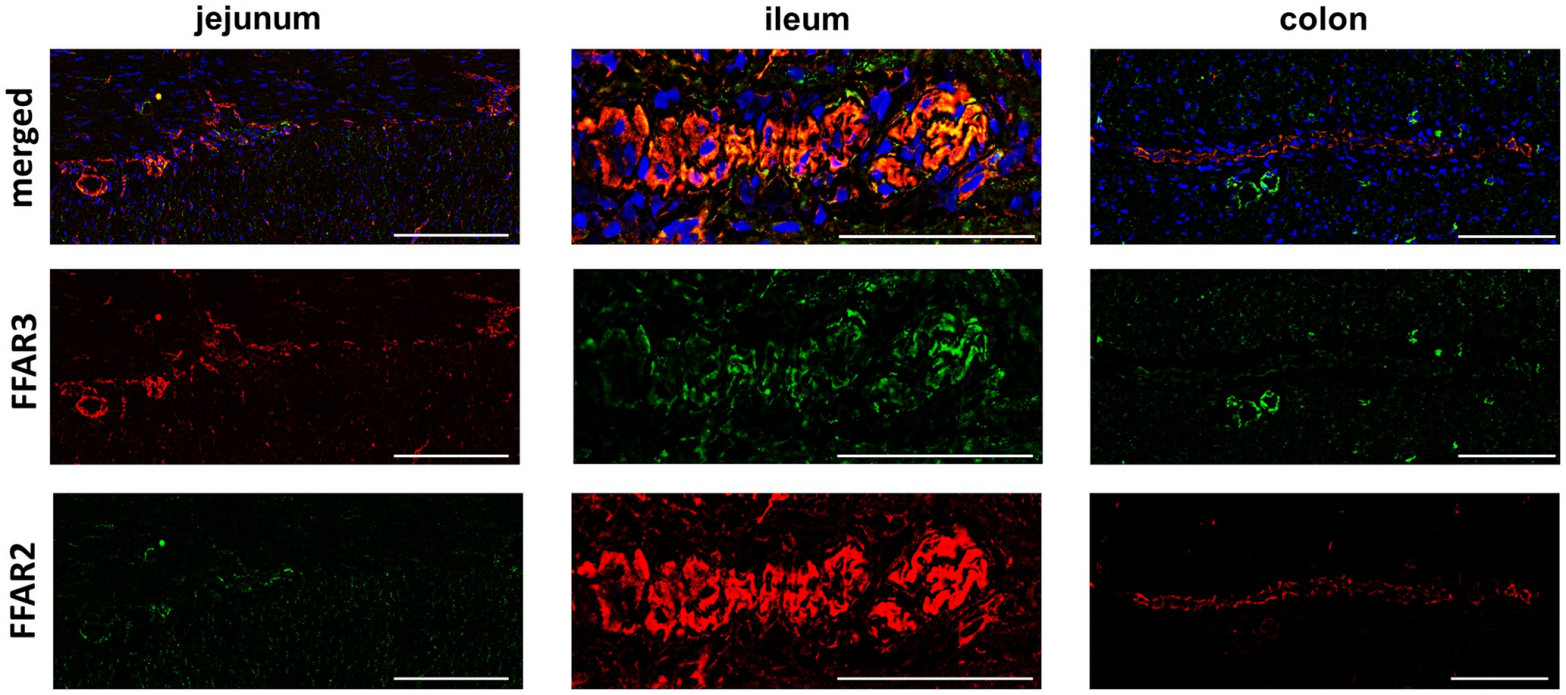
FFAR 2 and FFAR 3 in plexus myentericus. Gut sections (5 μm) of jejunum, ileum and colon were stained with anti-FFAR2 (red) and anti-FFAR3 (green) antibodies (3 replicates). Furthermore, DAPI (blue) was used for nuclei staining. Plexus myentericus is located between stratum circulare and stratum longitudinale and was double positive for FFAR2 and FFAR3 in all three gut segments [jejunum: bar = 200μm; ileum: bar = 100μm; colon: bar = 100μm].

Between the *stratum circulare* and *stratum longitudinale* of the *tunica muscularis* a double stained plexus myentericus was observed independent of the gut segment (Fig 5). Here, FFAR2 was highly expressed in the plexus myentericus and FFAR3 showed a weak expression.

#### Immune cells

**Fig 6:**
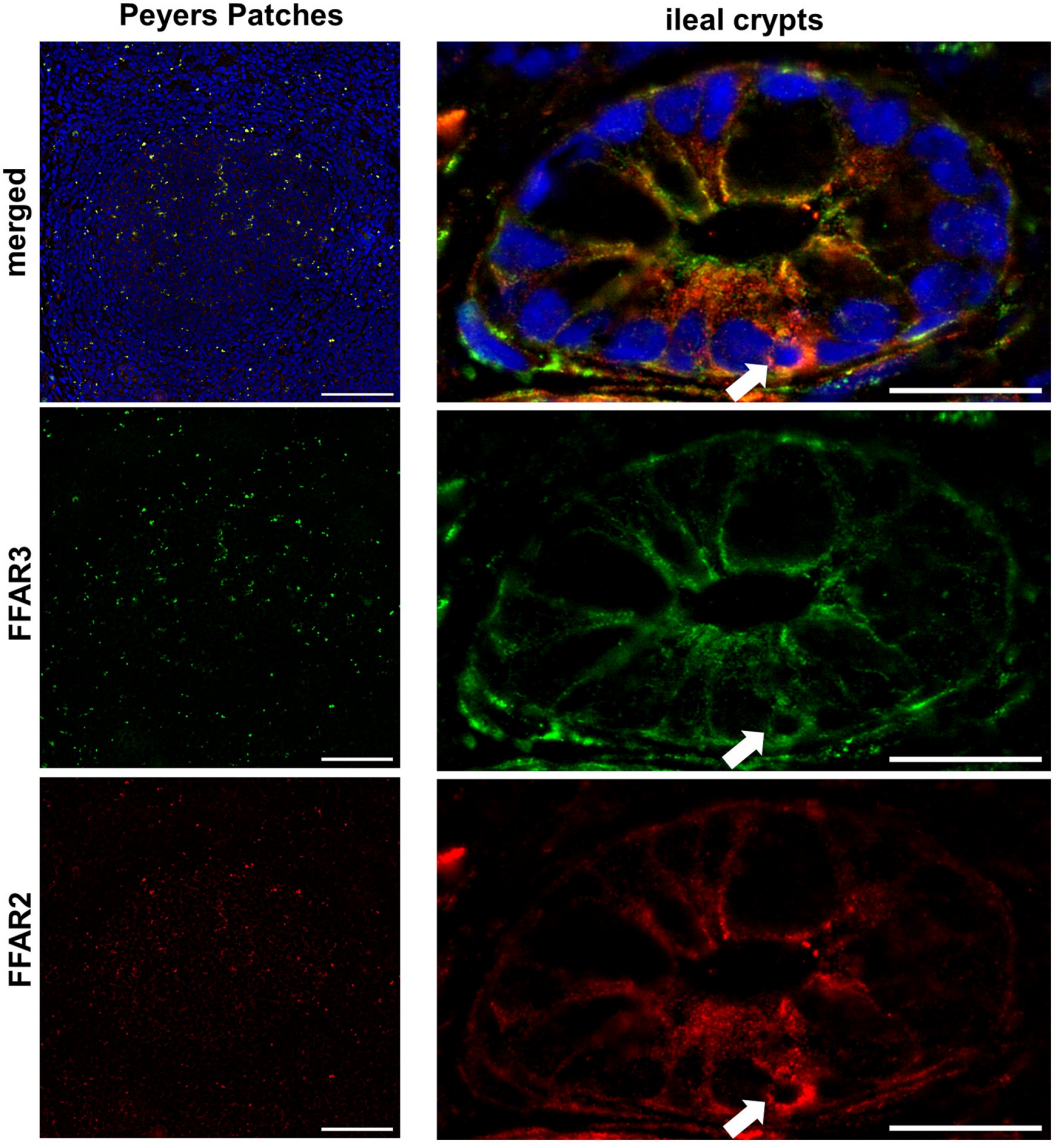
FFAR2 and FFAR3 in immune cells. Immunofluorescence labelling was performed with 5μm frozen gut section and were stained with an anti-FFAR2 (red) and anti-FFAR3 (green) antibody. Nuclei were marked with DAPI (blue). We observed cells with a strong autofluorescent granula, which were distributed all over the Peyer`s patches. Lymphocytes with a weak expression of FFAR3 were located in the germinal centre and mantle zone of peyer`s patches [bar = 100μm]. FFAR2 was less expressed in the mantle zone than in the germinal centre. Furthermore, double positive enterocytes but also small double-positive cells with a round nucleus between the enterocytes were detected [bar = 20μm]. Such positive cells were also found in the lamina epithelialis mucosae of the villi and were identified as lymphocytes.

A weak expression of FFAR3 on lymphocytes in the germinal centre and mantle zone of peyer`s patches were observed (Fig 6; S1–S11 Figs). FFAR2 was less expressed in the mantle zone than in the germinal centre. In addition, cells with a strong autofluorescent granula were obtained, which were distributed all over the Peyer’s patches.

In ileal crypts, we observed double-positive enterocytes but also small double-positive cells with a round nucleus (Fig 6). Such small double-positive cells were also found in the *lamina epithelialis mucosae* of the villi and were identified as lymphocytes.

### Confocal analysis of FFAR2 and FFAR3 in IPEC-J2

**Fig 7:**
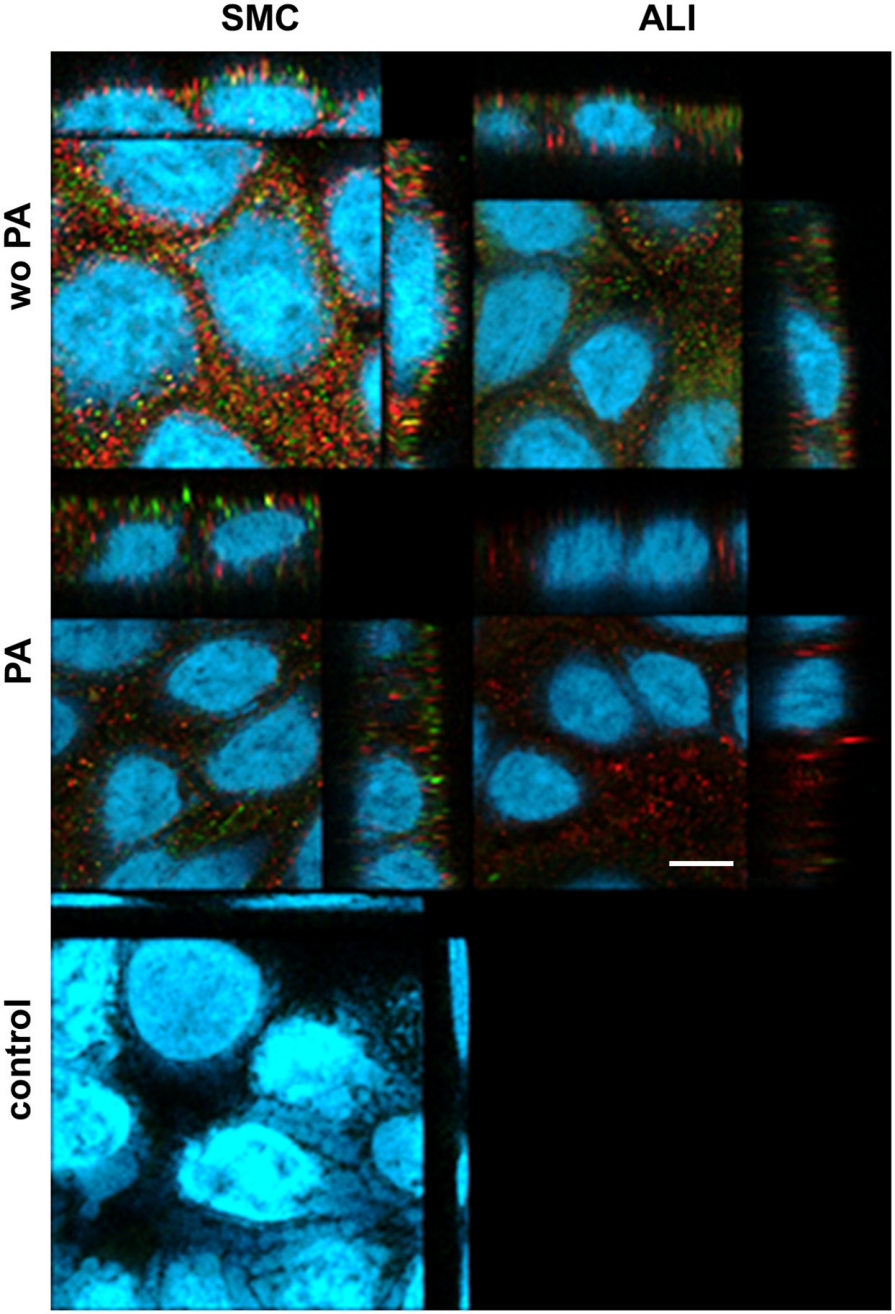
Confocal microscopy of FFAR2 and FFAR3 in IPEC-J2. IPEC-J2 cells were stained with FFAR2 (red), FFAR3 (green) and with DAPI (blue) for nuclei staining and analysed with confocal microscopy (N = 3]. No differences between the treatment groups were found in the distribution of FFAR2 and FFAR3 in IPEC-J2 cells. Additionally, a control for unspecific binding of the secondary antibody was performed (= control). Here, all treatment groups of high glucose are exemplarily shown. Both receptors were observed in the cytoplasm independent of the treatment group. Furthermore, expression of both receptors varied strongly within the monolayer of the cells. [bar = 5 μm].

FFAR 2 (red) and FFAR3 (green; Fig 7) were expressed in IPEC-J2 cells. A cytoplasmatic and perinuclear staining of FFAR2 and FFAR3 within the cells but both receptors were not located at the plasma membrane of the cells. We found an inhomogeneous expression pattern with the monolayers.

### RNA and protein expression of FFAR2 and FFAR3

**Fig 8:**
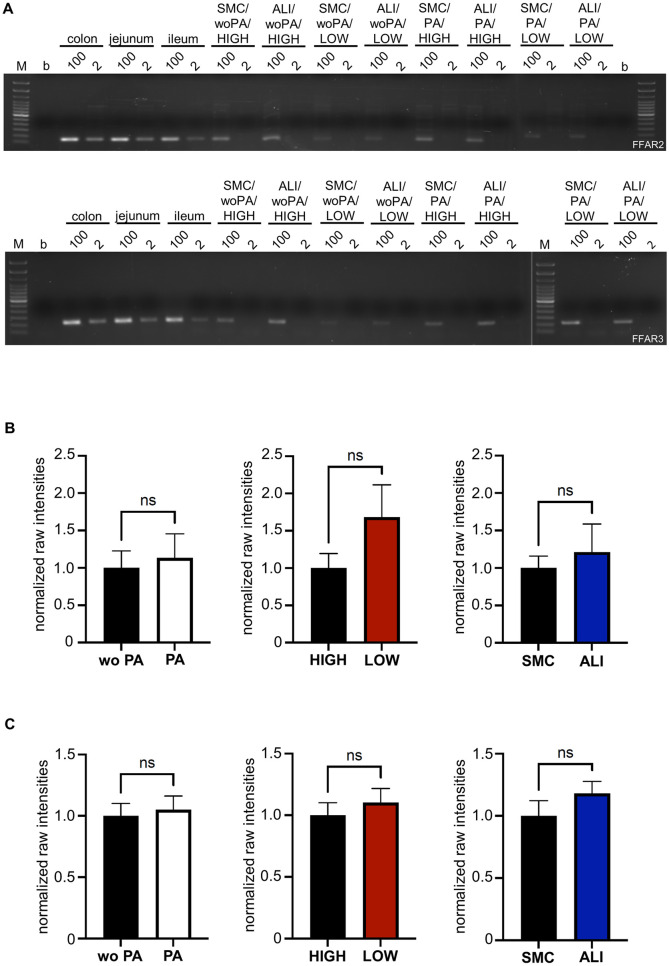
RNA (N = 5) and protein expression of FFAR2 and FFAR3 (N = 3). **(A)** 100 ng and 2 ng of DNA was used for RT-PCR of FFAR2 and FFAR3. Seven controls were performed: blank [b; no DNA], colon 100/2 ng, jejunum 100/2 ng, ileum 100/2 ng. IPEC-J2 samples of one experiment were exemplified: SMC/wo PA/HIGH 100/2 ng, ALI/wo PA/HIGH 100/2 ng, SMC/wo PA/LOW 100/2 ng, ALI/wo PA/LOW 100/2 ng, SMC/PA/HIGH) 100/2 ng, ALI/PS/HIGH 100/2 ng, SMC/PS/LOW 100/2 ng and (21/22) ALI/PS/LOW 100/2 ng. A strong expression of FFAR2 (100–200 bp) and FFAR3 (200–300 bp) was observed in all gut sections. In IPEC-J2 samples, 2 ng of DNA resulted in no detection of a band. **(B)** Western blot analyses of FFAR2 and **(C)** FFAR3 were quantitatively analysed. Therefore, raw intensities were normalized to the loading control β-actin. We found no differentially expression of FFAR2 and FFAR3 with the focus on the application of propionic acid, glucose or through the cultivation system.

RT-PCR war used for analysis of RNA expression and sample of gut sections (jejunum, ileum and colon) were used as positive control. 100 ng and 2 ng DNA were applied (Fig 8A). Therefore, transcription of RNA into DNA was performed via First Aid Reverse Transcription Reagent and DNA of gut sample were directly isolated. FFAR2 (100–200 bp) and FFAR3 (200–300 bp) was detected in all gut sample independent of used quantity. Contrary to gut segments, in IPEC-J2 samples a band was only observed with 100 ng used DNA content. qPCR with 2 ng RNA could not be evaluated.

Furthermore, analyses of protein expression demonstrated a stable expression of FFAR2 (Fig 8B) and FFAR3 (Fig 8C) in all treatment groups. All 3 comparisons (wo PA vs. PA, HIGH vs. LOW; SMC vs ALI) resulted in no significant differences (S12 and S13 Figs).

### Epithelial integrity

**Fig 9:**
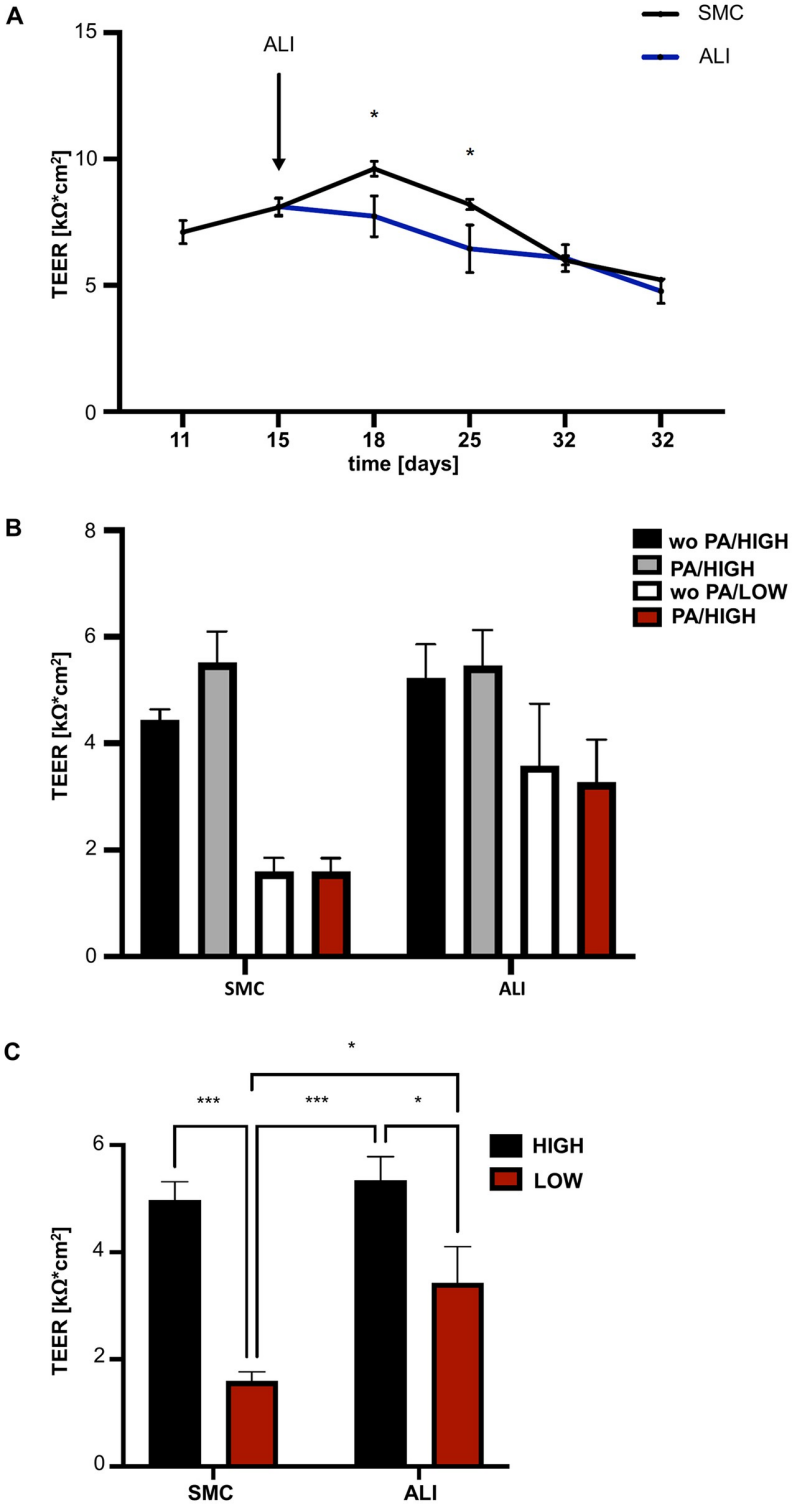
Transepithelial electrical resistance (TEER) (N = 6). **(A)** During the cultivation of SMC and ALI we observed significantly differences on day 18 (SMC: 9.61 kΩ*cm^2^; ALI: 7.73 kΩ*cm^2^; p<0.05) and day 25 (SMC: 8.19 kΩ*cm^2^; ALI: 6.44 kΩ*cm^2^; p<0.05) ALI cultures showed significantly decreased values compared to SMC. **(B)** On day 36, medium was changed to w/wo glucose and/or w/wo PA. After 24h of treatment, TEER measurement was performed. A main effect with the focus on culture system (SMC/ALI) and glucose concentration (HIGH/LOW) was observed but no effect of PA was detected. High values were detected with high glucose (SMC/wo PA/HIGH: 4.4 k*Ωcm^2^; SMC/PA/HIGH: 5.51 kΩ*cm^2^; ALI/wo PA/HIGH: 5.23 kΩ*cm^2^; ALI/PA/HIGH: 5.46 kΩ*cm^2^) and lower values with low glucose (SMC/wo PA/LOW: 1.59 kΩ*cm^2^; SMC/PA/LOW: 1.59 kΩ*cm^2^; ALI/wo PA/LOW: 3.58 kΩ*cm^2^; ALI/PA/LOW: 3.27 kΩ*cm^2^. **(C)** For further analysis data were consolidated by combining data into 4 groups (SMC/HIGH; SMC/LOW; ALI/HIGH; ALI/LOW). Significantly lower values were found with low glucose in the comparison of SMC/HIGH vs. SMC/LOW (p<0.05) and between ALI/HIGH vs. ALI/LOW (p<0.05). Furthermore, significantly lower values were detected in SMC/LOW compared to ALI/LOW (p<0.05).

Transepithelial electrical resistance was measured during the 36 days of cultivation. Here, we observed on day 18 (SMC: 9.61 kΩ*cm^2^; ALI: 7.73 kΩ*cm^2^; p<0.05) and day 25 (SMC: 8.19 kΩ*cm^2^; ALI: 6.44 kΩ*cm^2^; p<0.05; Fig 9A) significantly decreased TEER values during ALI cultivation. On day 36, medium was changed to w/wo glucose and/or w/wo PA. After 24h of treatment, TEER measurement was performed (Fig 9B). A main effect with the focus on culture system (SMC/ALI) and glucose concentration (HIGH/LOW) was observed but no effect of PA was detected (S1 Table). High values were detected with high glucose (SMC/wo PA/HIGH: 4.4 k*Ωcm^2^; SMC/PA/HIGH: 5.51 kΩ*cm^2^; ALI/wo PA/HIGH: 5.23 kΩ*cm^2^; ALI/PA/HIGH: 5.46 kΩ*cm^2^) and lower values with low glucose (SMC/wo PA/LOW: 1.59 kΩ*cm^2^; SMC/PA/LOW: 1.59 kΩ*cm^2^; ALI/wo PA/LOW: 3.58 kΩ*cm^2^; ALI/PA/LOW: 3.27 kΩ*cm^2^. For further analysis data were consolidated by combining data into 4 groups (SMC/HIGH; SMC/LOW; ALI/HIGH; ALI/LOW; Fig 9C). Significantly lower values were found with low glucose in the comparison of SMC/HIGH vs. SMC/LOW (p<0.05) and between ALI/HIGH vs. ALI/LOW (p<0.05). Furthermore, significantly lower values were detected in SMC/LOW compared to ALI/LOW (p<0.05).

### Analyses of ENO1 and NDUFA4 on RNA-level

**Fig 10:**
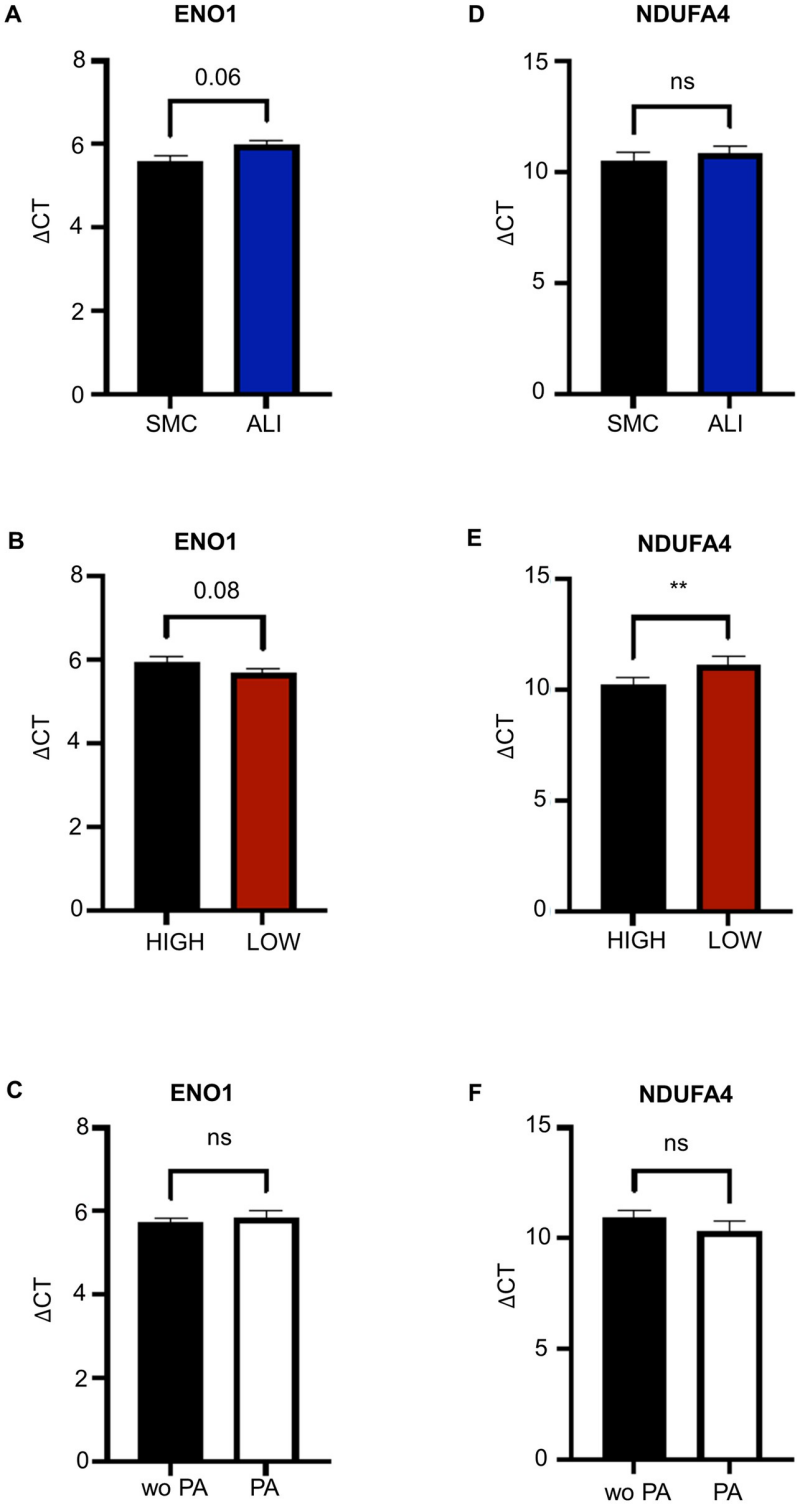
Analysis of ENO1 and NDUFA4 on mRNA-level in IPEC-J2 (N = 5). Two important genes of the metabolism, ENO1 and NDUFA4, were analysed via qPCR. (A) Decreased ENO1-mRNA-expression was found in ALI-groups in the comparison the SMC-groups (p = 0.06). (B) In contrast, increased ENO1-mRNA-expression was observed in the LOW-glucose-groups compared to the HIGH-glucose-groups (p = 0.08). (C) ENO1 showed no differences in the comparison of with/without propionic acid. (D) When comparing SMC/ALI but also with/without PS (F), no significant differences were found. (E) A significant decrease of NDUFA4-mRNA was observed in LOW-glucose-groups compared to HIGH-glucose-groups (Fig 8E; p<0.01**).

Two important gene of the metabolism were analysed via qPCR. Decreased ENO1-mRNA-expression was found in ALI-groups in the comparison the SMC-groups (Fig 10A; p = 0.06). In contrast, increased ENO1-mRNA-expression was observed in the LOW-glucose-groups compared to the HIGH-glucose-groups (Fig 10B; p = 0.08). ENO1 showed no differences in the comparison of with/without propionic acid (Fig 9C). When comparing SMC vs. ALI (Fig 8D) but also with and without PS (Fig 10F), no significant differences were found. A significant decrease of NDUFA4-mRNA was observed in LOW-glucose-groups compared to High-glucose (Fig 10E; p<0.01**).

### Functional analysis of mitochondria

**Fig 11:**
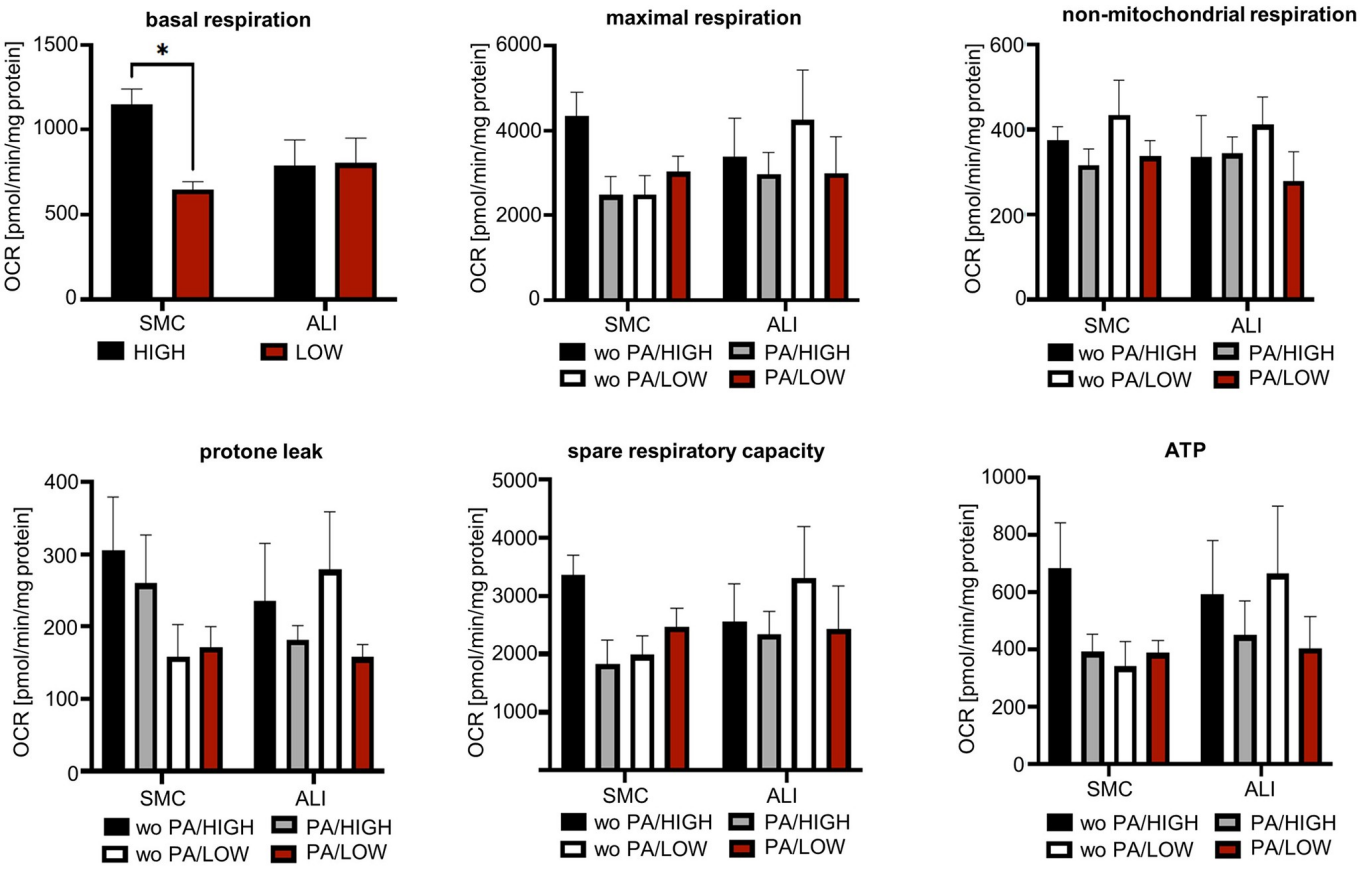
OCR—Oxygen consumption rate. The OCR is represented as mean values normalized to protein content (N = 3/4). The error bar represents the standard error of the mean values. Various respiratory parameters were examined such as maximal respiration, non-mitochondrial respiration, proton leak, spare respiratory capacity and ATP. The evaluation of the basal respiration was also carried out with a three-factor ANOVA. Here, a significant interaction effect between cultivation and glucose content was obtained (p<0.05). The dataset was consolidated and a two-factor ANOVA was performed. This resulted in an ordinal interaction effect but also in a significant main effect of glucose content (p < 0.05).

The evaluation of oxidative consumption rate (OCR) included the parameters of basal respiration, maximal respiration, ATP-associated respiration, respiratory reserve capacity, non-mitochondrial respiration and proton leak (Fig 11). With the regard to basal respiration, a significant interaction effect between the cultivation (SMC vs. ALI) and the glucose content (HIGH/LOW) could be determined (p<0.05; S2 and S3 Tables). Based on this result, the data set was consolidated, and a two-factorial ANOVA was performed. Here, a significant main effect of the glucose content was found (p<0.05, Fig 11). Furthermore, a significant difference between SMC/HIGH and SMC/LOW was observed within the Post-hoc-tests (p<0.05). No significant differences were between the groups in the following parameters: maximal respiration, ATP-associated respiration, respiratory reserve capacity, non-mitochondrial respiration and proton leak (Fig 11).

**Fig 12:**
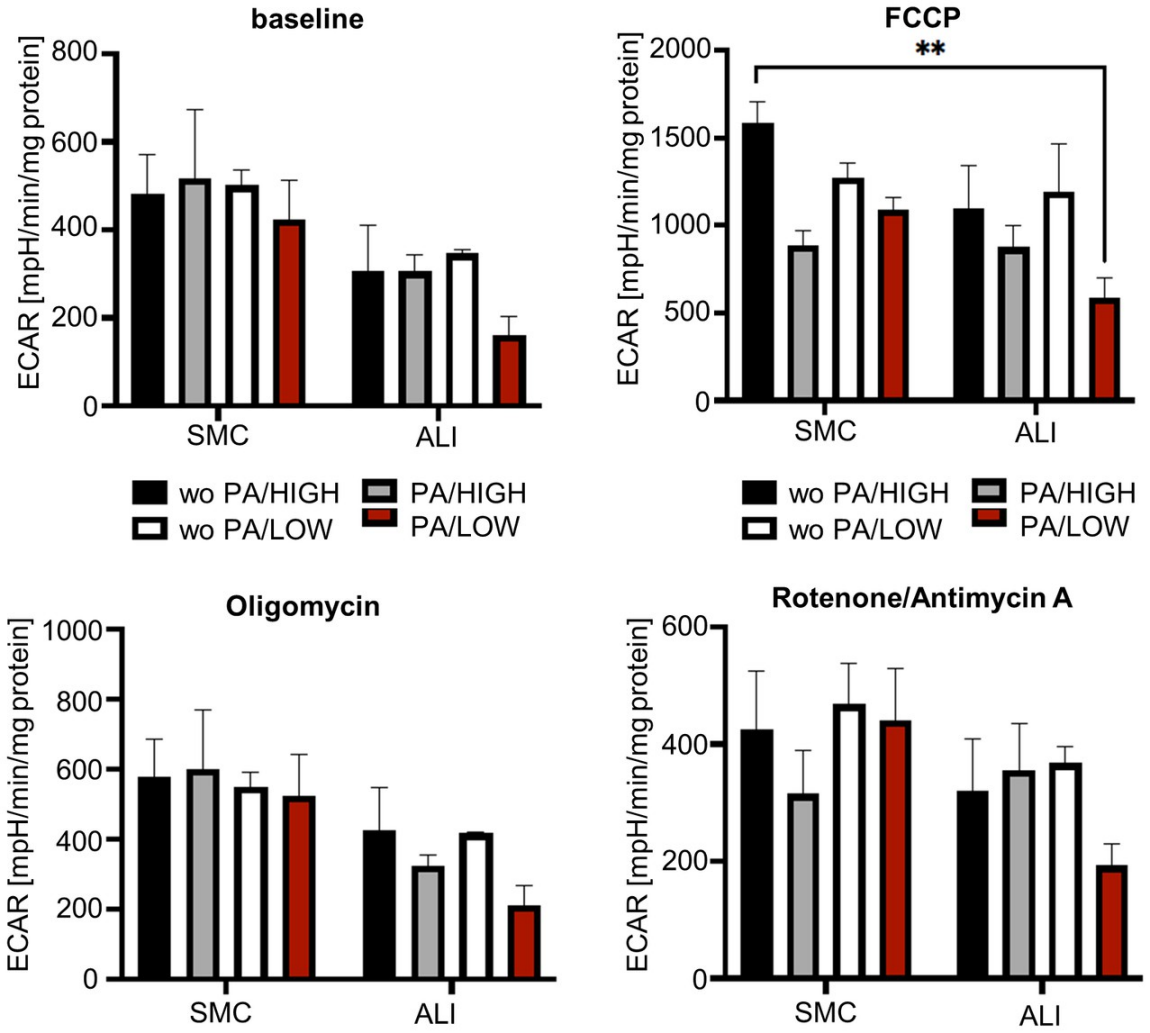
ECAR—Extracellular acidification rate. The figure shows the acidification rate recorded with the Mitostress test, which includes the mean values normalized to protein content (N = 3). The error bar represents the standard error of the mean values. The evaluation of the ECAR with a three-factor ANOVA resulted in a significant main effect of the cultivation (SMC vs. ALI; p<0.01) within the “Baseline”. Here, all treatment groups of ALI cultivation showed a lower ECAR compared to CON cultivation. The same result was obtained after injection of FCCP and Oligomycin (p<0.05). After FCCP injection a significant main effect was found with the addition of propionic acid. A significant lower ECAR was observed in ALI/PA/LOW compared to SMC/woPA/HIGH with a Tukey-Post-hoc-test.

Furthermore, extracellular acidification rate was examined (Fig 12, S4 Table). When looking on the baseline (p<0.01) but also after oligomycin injection (p<0.05), a significant main effect could be found. Here, all ALI treatment groups showed consistently lower ECAR-values (Fig 12). After FCCP injection, cultivation (SMC vs. ALI; p<0.05) and addition of propionic acid (PA; p<0.1) resulted in a significant main effect (Fig 12). The analysis of the ECAR values after rotenone/antimycin A injection resulted in no significant results.

## Discussion

### Distribution of FFAR2 and FFAR3 in the porcine gut

The receptors of short fatty acids FFAR2 and FFAR3 have hardly been researched in intestinal tissues of pigs up to date. In this work, we were able to detect for the first time FFAR2 and FFAR3 in different gut sections of *sus scrofa* by immunofluorescence staining. The distribution of FFAR2 and FFAR3 along the crypt-villus axis has been uniformly described. In human and murine ileum and colon, there is a strong expression in the luminal epithelium, which becomes weaker towards the crypts [16–18]. This trend could be confirmed in colon for *sus scrofa*. Here, a strong staining of FFAR2 and FFAR3 of the luminal epithelial cells was observed, which decreased from the luminal side to the bottom of the crypts. In jejunum and ileum, the observations varied greatly between the individual replicates. To date, little is known about the expression of these receptors in the intestine. Some studies have shown that FFAR2 and FFAR3 expression is influenced by their ligands. In goats, the addition of SCFA to cell culture media has been shown to upregulate FFAR2 and FFAR3 expression on protein-level and mRNA-level [19]. An increased expression of FFAR2 and FFAR3 was also observed in pigs treated with Lactobacillus reuteri. The authors speculate that this leads to increased butyrate formation, which in turn leads to increased expression of FFAR2 and FFAR3 [20]. If the expression is concentration dependent, expression should be greater luminally than in the crypts. A decrease along crypt-villus-axis was particularly clearly observed in the colon. The SCFAs were metabolized by colonocytes, which produce their energy by butyrate oxidation [21]. In addition, some authors supposed that the intestinal stem cell niche is protected based on the degradation of SCFAs towards the cryptal bottom [22]. Moreover, there are also differences in the bacterial colonization towards the crypt-villus axis and the outcome of this is a differentially production of SCFAs [23].

In addition, we observed a cytoplasmatic staining and a strong staining of the apically plasma membrane of enterocytes in the colon. The research group of Tazoe et al. (2009) discovered that in the human colon FFAR3 is expressed within the mucosa on enterocytes, as well as enteroendocrine cells [17]. Furthermore, they described the receptor distribution within enterocytes similar to us, showing cytoplasmic staining in the endoplasmic reticulum and Golgi complex, as well as in the apical cell body, but not in the membrane. So, they concluded that FFAR3 is transported via the ER and Golgi complex to the apical cell membrane. We obtained a strong expression of FFAR2 and a moderate FFAR3 signal below FFAR2. Thus, both receptors appear in the apical membrane as two separate lines in the colon. This was not observed in the jejunum and ileum. The brush border lies on the terminal web. This represents a network of filaments. The roots of the microvilli are anchored in this network via myosin and spectrin [24]. Ultrastructural analyses show that apically to the terminal web, at the boundary to the beginning luminal part of the microvilli, there is a line on which the microvilli rest [25]. This is called intermicrovillous membrane and thus represents the boundary to the terminal web. The broad FFAR3 positive seam corresponds most closely to the region of the terminal web while the overlying seam of FFAR2 corresponds more closely to the intermicrovillous boundary. Additional histochemical and ultrastructural investigations are required to confirm this classification.

In all sections of the gut FFAR2 and FFAR3 was obtained in the intestinal smooth muscle cells of the stratum circulare and stratum longitudinale. FFAR2 was only weakly expressed in the colon, or no expression was observed in the smooth muscle cells. Early studies in propionate showed that an application in veins leads to a contraction in the ileum of rats [26]. Yajima induced a contraction also in colon of rats due to luminal application of propionate but not due to serosal application [27]. Furthermore, these contractions were blocked by atropine, which leads to the conclusion that they were acetylcholine mediated. Yajima hypothesized that SCFAs led to an acetylcholine-release which was mediated by enterocytes [27]. However, Cherbut and colleagues showed a SCFA induced contraction of isolated muscle stripes of the terminal ileum, which is independent of an intact tunica mucosa [28]. In addition, Yajima and colleagues were later able to record that there are ACh producing cells in the murine colon crypts, which are activated by propionate and acts as a sensor for propionate. It is not clear how they can recognize propionate. It is supposed that FFAR2 and/or FFAR3 play a role in the recognition [29].

FFAR3 was identified in neurones of sympathetic, parasympathetic and somatic ganglions as well as nerve processes of the portal vein wall [30–32]. Moreover, FFAR3 was observed in ganglion cells of the intestine and nerve processes of the plexus myentericus, submucosus and mucosus but no FFAR2 was obtained [33–35]. In this study, a strong expression of FFAR2 and a moderate expression of FFAR3 was illustrated in the porcine enteric nerve system. Ganglion cells were homogeneously stained and no single cell somata could be defined. Kaji and colleagues assume a negative feedback mechanism. Hence, the gut activity is increased due to a short-term food intake and therefore due to the increase of SCFAs but the excessively intake of SCFAs reduces the activity as the SCFAs in the blood inhibit the enteric nerve system by FFAR3 [34, 35].

Already in 2003, FFAR2 and FFAR3 was investigated in vessels and only FFAR3 was obtained in endothelial cells of arteries but not in in veins or lymphatic vessels [36]. We detected FFAR2 in vascular smooth muscle cells of arteries, veins and lymphatic vessels but also in endothelial cells of ileal vessels. FFAR3 was strongly expressed on endothelial cells of veins and lymphatic vessels but also in ileal arteries and vascular smooth muscle cells. In previous studies, it was shown that acetate acts as a vasodilatator [37]. Furthermore, in FFAR3 knockout mice an isolated systolic hypertension was found which confirm the role of FFAR3 as a regulator of the blood pressure [38].

### Expression and morphology of FFAR2 and FFAR3 *in vitro*

Both receptors were documented on protein- and mRNA level in IPEC-J2 and the effect of glucose content and cultivation model was examined. A detection of FFAR2 and FFAR3 with qPCR was not possible perhaps due to small content of applied mRNA of 2 ng. However, a RT-PCR was performed and 2ng and 100ng of cDNA was applied and a signal was detected. In addition, both receptors were obtained with western bot analyses and immunofluorescence staining. FFAR3 showed a double protein band. This was described by other groups and a posttranslational modification is the underlying mechanism of these bands [17, 39]. Moreover, there is a possible correlation between glucose content and FFAR2/3 expression, because we found a higher expression of FFAR2 in CON groups with low glucose content. Perhaps a lower glucose content induces an increase of the differentiation of the cells. Furthermore, we investigated our cells via immunofluorescence staining and observed both receptors but not in the apical membrane of the cells but more in the cytoplasm of the cells. Tazoe and colleagues described a cytoplasmatic staining in the endoplasmatic reticulum and Golgi complex as well as at the apical side of the enterocytes but not in the membrane in vivo [17]. They postulated as already mentioned, that FFAR3 is transported via ER and Golgi complex to the apical membrane. Furthermore, IPEC-1 and IPEC-J2 cells are both non-transformed and had their origin in newborn piglets [40]. It is supposed that they have perhaps a stem cell character. This would explain the low expression of FFAR2 and FFAR3, because we found a decrease of both receptors from the top of the villus to the bottom of the crypts.

### Effect of propionic acid on the epithelial integrity

The effect of butyrate is well-documented in Caco-2 and other cell lines. An incubation with butyrate causes an up-regulation of tight junction proteins like ZO-1, ZO-2, CLDN-1 and CLDN-2, which was quantified with TEER measurements [41–44]. There is a lack of information regarding to propionate and IPECs or rather the effect of SCFA in pigs. No effect was observed on epithelial integrity in IPEC-J2 after an incubation of 24h. In contrast, an incubation with butyrate increases TEER-values and tight junction proteins [45], but they incubated for only 8 days not for 24h.

Furthermore, we suggest that TEER values will increase with the extension of incubation time and/or an increase of propionate concentration. Suzuki and Kleuskens showed an increase of the epithelial integrity within their studies but they used higher concentration of propionate: 20 mM and 10 mM instead of 4.87 mM [46, 47].

### Effects on metabolism

Short-chain-fatty acids such as acetate, propionate and butyrate are able to influence cellular metabolism in different ways. Hu et al. [48] showed that one possibility is the activation of FFAR2. Using a shRNA knockdown of FFAR2, the authors were able to demonstrate that acetate stimulates mitochondrial biogenesis via FFAR2 in plurivacuolar adipocytes of brown adipose tissue. The reduced extracellular acidification rate can mainly be explained by a propionate-induced steering of the metabolism away from the acidifying glycolysis towards OXPHOS. However, it should be noted that extracellular acidification is not only influenced by the produced lactate due the glycolysis, but also by CO_2_ from the citrate cycle. However, this makes up a small proportion of the total ECAR [49, 50]. Based on the ECAR results, an increase in OCR would be expected, but this was not the case. The reason is probably to be found in the validity of the OCR data. In addition to high intra- and inter-trial variability, which was also observed by Qi et al. [51], a number of faulty technical replicates were also found. These are presumably caused by air pockets in the medium or in the membranes, which were released after starting the experiment. In our experiments, the air entrapment can probably be explained by the use of membranes as culture underground. Membranes were removed from the plastic frame on the day of the experiment and placed in the assay plate. The transfer of the membranes, but also the subsequent insertion of the islet capture screens can cause small air bubbles within the wells.

The decrease in glucose concentration should trigger the metabolism more towards OXPHOS, so that ENO1 is down-regulated in the LOW-group. However, the RNA-expression of ENO1 showed a trend of up-regulation in the LOW-group compared to the HIGH-group. Xu and co-workers observed an up-regulation of ENO1 on RNA- and protein-level with increased glucose concentration (5.5–25 mM) [52]. A possible reason for the contrary expression of ENO1 could be related to the acute reduction of glucose. This is possibly associated with a subsequent AMPK activation. This in turn results in a promotion of glycolysis. Various studies have shown that ALI cultures promotes OXPHOS [6, 7]. An important regulator of the metabolic adaption is the heterodimeric transcription factor HIF-1, which is an oxygen sensor in the cell [53]. HIF-1 consists of 2 subunits HIF-1α and HIF-1β. The β-subunit is expressed constitutively, while the α-subunit is mainly present under hypoxic conditions in a stable state [54, 55]. The oxygen-dependent protein expression of HIF-1α is realized by prolyle hydroxylases. Under normoxia, hydroxylation of prolyle residues occurs, leading to the degradation of HIF, which is mediated by the von-Hippel-Lindau-protein [56, 57]. Thus, HIF-1α is stabilized under hypoxia and can function as transcription factor. The HIF-regulated genes influence metabolism in complex ways. This results in a promotion of glycolysis and an inhibition of OXPHOS. Glycolysis is promoted by the transcription of the enzymes which are involved in glycolysis, such as HK2, ENO1, LDHA and GLUT1/3 and MCT4 [58, 59]. We detected a down-regulation of ENO1 with the focus on cultivation (ALI vs. CON) in our ALI-group, which is consistent with the results of Klasvogt et al [7] and the hypothesis that ALI promotes OXPHOS.

NDUFA4 shows reduced mRNA expression level in the low group, which was not to be expected. It must be pointed out, there are only a few studies on NDUFA. It is known, that NDUFA is inhibited by microRNA miR-210. This in turn is itself up-regulated under oxygen and/or glucose deprivation [60]. Interestingly, however, an isolated increase of glucose concentration, e.g. 30 mM, also leads to increased expression of miR-210 [61]. Comparable experiments with low glucose concentration, as shown here, have not been carried out yet.

## Conclusion

For the first time, the morphological expression of FFAR2 and FFAR3 was investigated in pig using immunofluorescence. The expression pattern of human and murine epithelial cells in the jejunum, ileum and colon was confirmed. Furthermore, new findings could be obtained. Both receptors were detected in smooth muscle cells of the intestinal musculature. Furthermore, the expression of FFAR2 and, to a lesser extent, FFAR3 was observed in the enteric nervous system of the jejunum, ileum and colon. In addition, FFAR2 was detected for the first time in smooth muscle cells of arteries, veins and lymphatic vessels. FFAR3, on the other hand, is strongly expressed by endothelial cells of veins as well as lymphatic vessels, and also by ileal arterial endothelial cells and vascular smooth muscle cells.

The observation of FFAR2 and FFAR3 in porcine enterocytes and further in vessels and plexus myentericus offers new starting points to investigate the interaction of the microbiome and the gut homeostasis. At the same time, the cell line IPEC-J2 provides the opportunity to engross the investigations of the impact of SCFAs on the metabolism. This can be realized with the combination of butyric acid, propionic acid and acetic acid and the extention of the incubation time.

## Supporting information

S1 Fig FFAR2 and FFAR3 distribution in a jejunal gut segment. Frozen sections (animal 1) of the jejunum were labelled with antibodies for FFAR2 (red) and FFAR3 (green). Additionally, DAPI (blue) war used for nucleus staining. bar [= 50 μm].
(TIF)

S2 Fig FFAR2 and FFAR3 distribution in an ileal gut segment. Frozen ileal sections (animal 1) were labelled with antibodies for FFAR2 (red) and FFAR3 (green). DAPI (blue) war used for nucleus staining. bar [= 50 μm].
(TIF)

S3 Fig FFAR2 and FFAR3 distribution in a jejunal gut segment. To investigate the distribution of FFAR2 and FFAR3, frozen jejunal sections (animal 2) were stained with antibodies for FFAR2 (red) and FFAR3 (green). Nucleus staining was performed with DAPI (blue). FFAR3 was more strongly expressed in enterocytes of the jejunum than FFAR2. This was also the case for smooth muscle cells in the tunica mucosa. bar [= 50 μm].
(TIF)

S4 Fig FFAR2 and FFAR3 distribution in an ileal gut segment. Using immunofluorescence labelling, distribution of FFAR2 (red) and FFAR3 (green) was made visible in frozen sections of ileum (animal 2). Furthermore, nuclei were stained with DAPI (blue). A strong FFAR3 expression was observed in endothelial cells of veins (§), enterocytes of the tunica mucosa and smooth muscle cells of the tunica muscularis. FFAR2 was overall weaker expressed than FFAR3. bar [= 50 μm].
(TIF)

S5 Fig FFAR2 and FFAR3 distribution in jejunal gut segment. A strong staining of FFAR3 (green) was observed in the endothelia of veins (§), in enterocytes of the tunica mucosa and in smooth muscle cells of the tunica muscularis in the jejunum of animal 3. bar [= 50 μm].
(TIF)

S6 Fig FFAR2 and FFAR3 distribution in ileal gut segment. Frozen sections of the ileum were stained with FFAR3 (green) and FFAR2 (red) and a strong labelling of FFAR3 was found in the enterocytes of the tunica mucosa. bar [50μm].
(TIF)

S7 Fig FFAR2 and FFAR3 distribution in jejunal gut segment. Frozen sections (animal 4) of the jejunum were labelled with antibodies for FFAR2 (red) and FFAR3 (green). Additionally, DAPI war used for nucleus staining. bar [= 50 μm].
(TIF)

S8 Fig FFAR2 and FFAR3 distribution in ileal gut segment. Using immunofluorescence labelling, distribution of FFAR2 (red) and FFAR3 (green) was made visible in frozen sections of ileum (animal 4). Furthermore, nuclei were stained with DAPI (blue). Endothelial cells of veins (§) showed a strong expression of FFAR3 (green) in contrast to FFAR2 (red). Additionally, enterocytes in the tunica mucosa expressed FFAR3 to a greater extent than FFAR2. Smooth muscle cells expressed only FFAR3 but not FFAR2. bar [= 50 μm].
(TIF)

S9 Fig FFAR2 and FFAR2 distribution in jejunal gut segment Frozen sections (animal 5) of the jejunum were labelled with antibodies for FFAR2 (red) and FFAR3 (green). Additionally, DAPI war used for nucleus staining. bar [= 50 μm].
(TIF)

S10 Fig FFAR2 and FFAR2 distribution in ileal gut segment. FFAR3 was strongly expressed in the tunica mucosa in contrast to FFAR2 (animal 5). Furthermore, a strong staining of endothelial cells was found in veins (§). The appearance of the cross sections of this vessel can lead to the conclusion that it could also be a lymphatic vessel but not a vein.
(TIF)

S11 Fig FFAR2 and FFAR3 expression in colon. The tunica mucosa of the colon showed both: a strong FFAR3 and a strong FFAR3 expression (animal 5). The smooth muscle cells in the tunica muscularis showed no or only a weak expression of FFAR2. Endothelial cells of veins (§) were strongly labelled with FFAR3 but not with FFAR2. bar [50μm].
(TIF)

S12 Fig Western blots of FFAR2. Western blot analyses were repeated (N = 3). Raw western blots of FFAR2 (predicted protein size 50 kDa, a-c) were shown with prestained page ruler. ß-actin (aa-cc) was used as loading control (38 kDa).
(TIF)

S13 Fig Western blots of FFAR3. Western blot analyses were repeated (N = 3). Raw western blots of FFAR3 (a-c) were shown with prestained page ruler. ß-actin (aa-cc) was used as loading control (38 kDa). Protein samples of FFAR2 and FFAR3 were loaded on separated gels due to the similarity of the protein size.
(TIF)

S1 Table Three-way ANOVA of TEER values on day 37. Main and interaction effects are shown in the table. The glucose content and cultivation as ALI or SMC showed significant effects on TEER values. The results based on 6 independent experiments.
(DOCX)

S2 Table Three-Way ANOVA of the baseline OCR. A three-way ANOVA was performed to analyse the OCR of the baseline. A significant interaction effect was found between cultivation and glucose (N = 3).
(DOCX)

S3 Table Two-way ANOVA of the baseline OCR. A new ANOVA (two-way) was performed under consolidation of the propionate group. A significant main effect of the cultivation (CON vs. ALI) was observed but also a significant interaction effect between cultivation and glucose content.
(DOCX)

S4 Table Three-way ANOVA of the baseline ECAR. A three-way ANOVA was performed with the baseline values of the ECAR (N = 3).
(DOCX)
