# Supplementary figures and images for: Modeling of culture conditions by culture system, glucose and propionic acid and their impact on metabolic profile in IPEC-J2
Source: PLoS One. 2024 Jul 18;19(7):e0307411. doi: 10.1371/journal.pone.0307411 (PMC11257281; doi:10.1371/journal.pone.0307411)

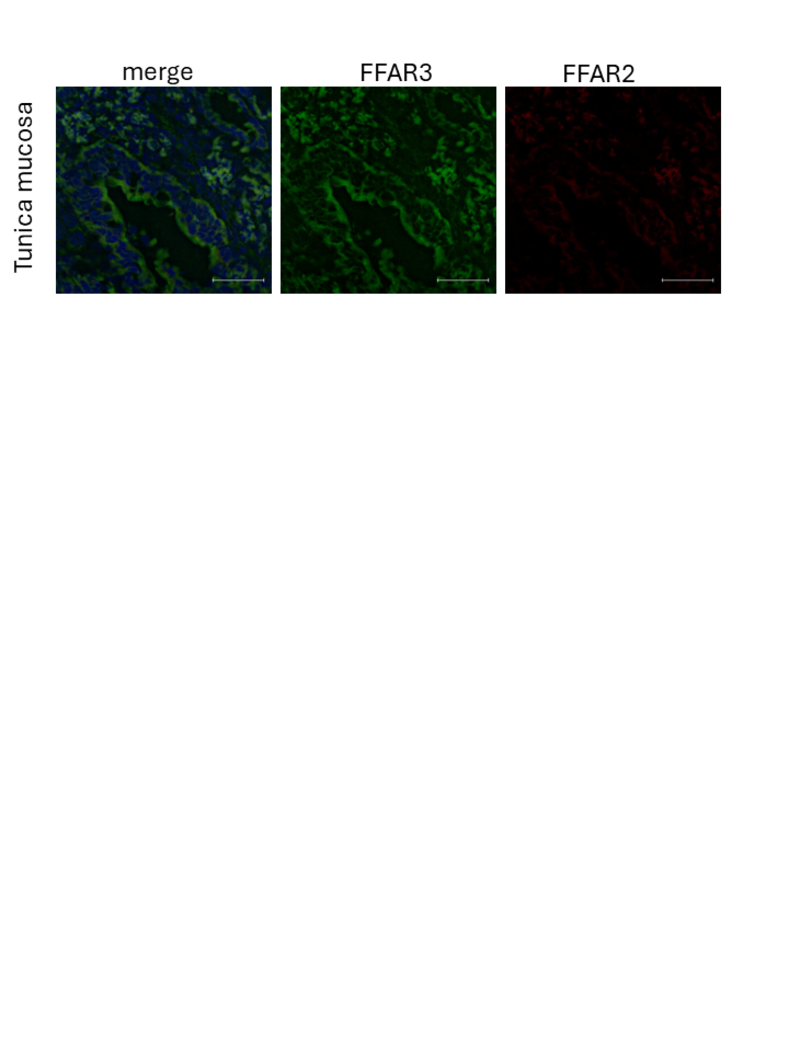

Supplement: S1 Fig — Frozen sections (animal 1) of the jejunum were labelled with antibodies for FFAR2 (red) and FFAR3 (green). Additionally, DAPI (blue) war used for nucleus staining. bar [= 50 μm]. (TIF) [file pone.0307411.s001.tif]

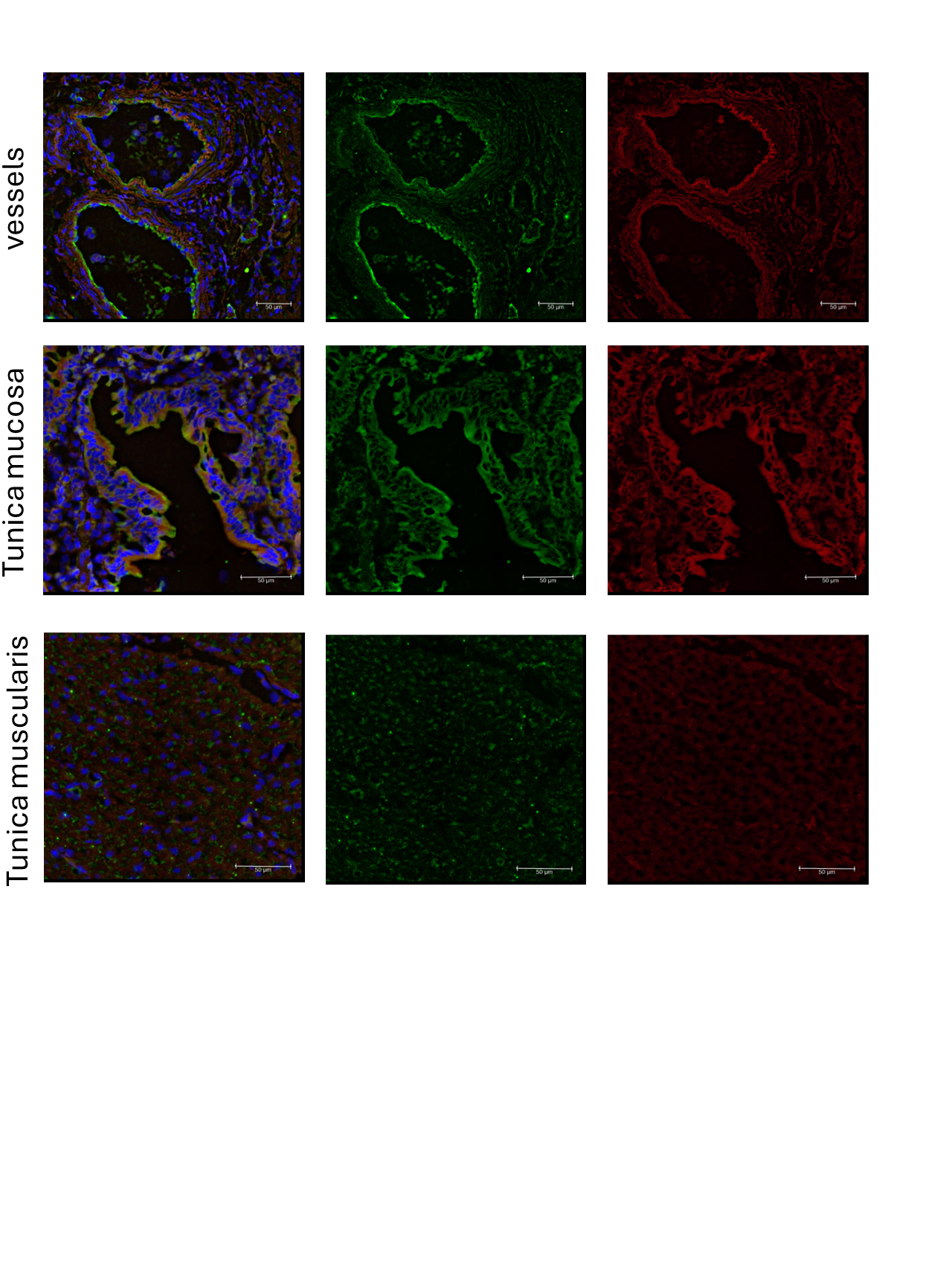

Supplement: S2 Fig — Frozen ileal sections (animal 1) were labelled with antibodies for FFAR2 (red) and FFAR3 (green). DAPI (blue) war used for nucleus staining. bar [= 50 μm]. (TIF) [file pone.0307411.s002.tif]

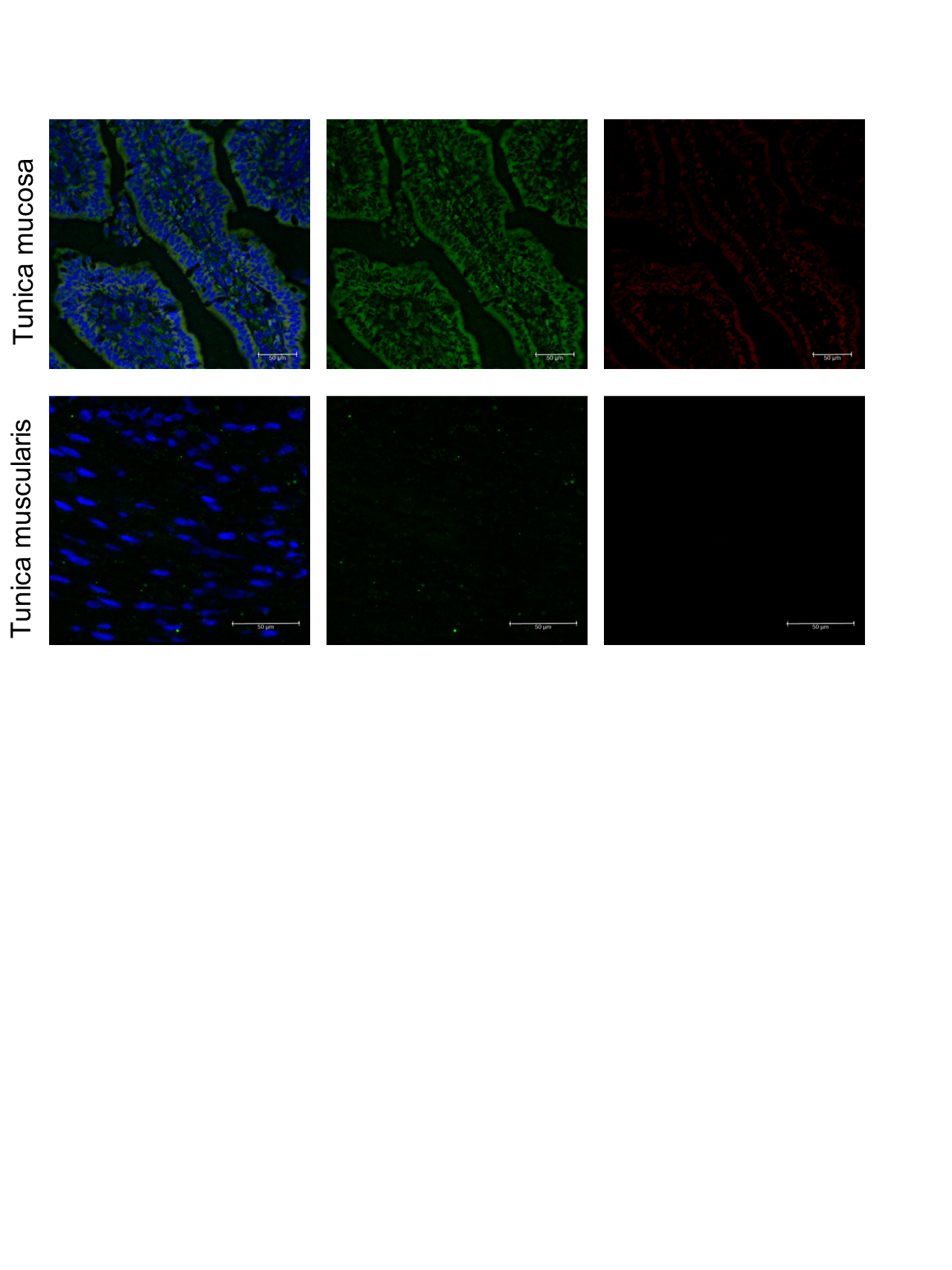

Supplement: S3 Fig — To investigate the distribution of FFAR2 and FFAR3, frozen jejunal sections (animal 2) were stained with antibodies for FFAR2 (red) and FFAR3 (green). Nucleus staining was performed with DAPI (blue). FFAR3 was more strongly expressed in enterocytes of the jejunum than FFAR2. This was also the case for smooth muscle cells in the tunica mucosa. bar [= 50 μm]. (TIF) [file pone.0307411.s003.tif]

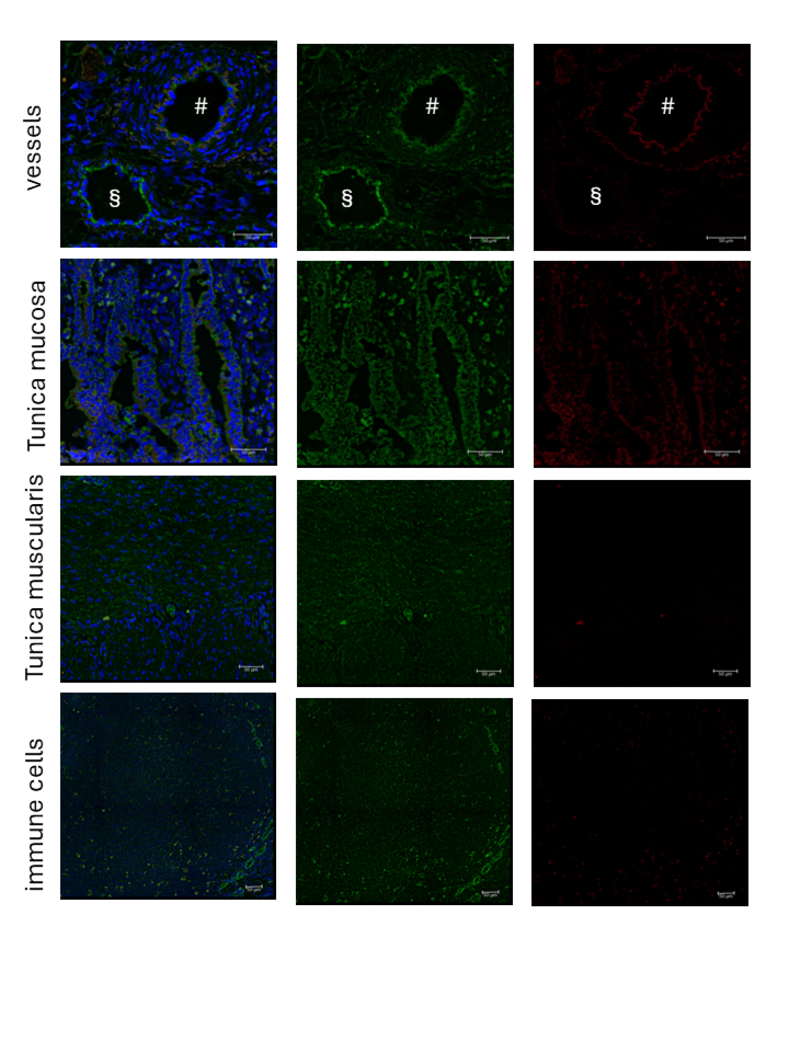

Supplement: S4 Fig — Using immunofluorescence labelling, distribution of FFAR2 (red) and FFAR3 (green) was made visible in frozen sections of ileum (animal 2). Furthermore, nuclei were stained with DAPI (blue). A strong FFAR3 expression was observed in endothelial cells of veins (§), enterocytes of the tunica mucosa and smooth muscle cells of the tunica muscularis. FFAR2 was overall weaker expressed than FFAR3. bar [= 50 μm]. (TIF) [file pone.0307411.s004.tif]

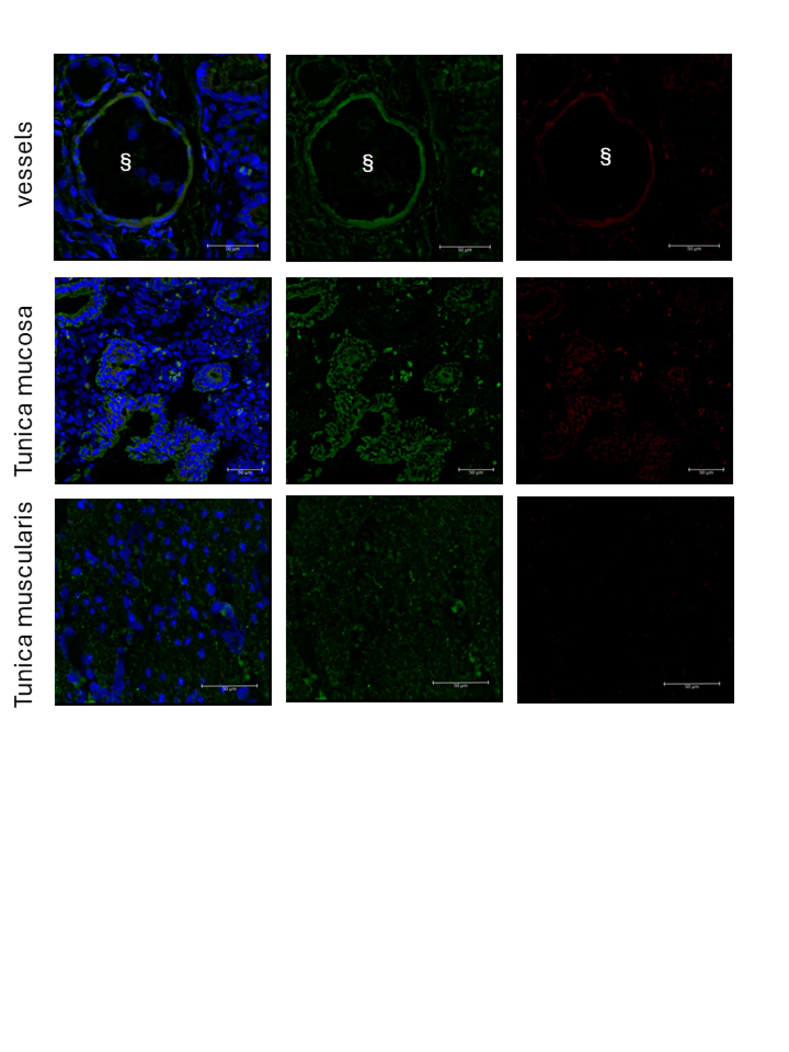

Supplement: S5 Fig — A strong staining of FFAR3 (green) was observed in the endothelia of veins (§), in enterocytes of the tunica mucosa and in smooth muscle cells of the tunica muscularis in the jejunum of animal 3. bar [= 50 μm]. (TIF) [file pone.0307411.s005.tif]

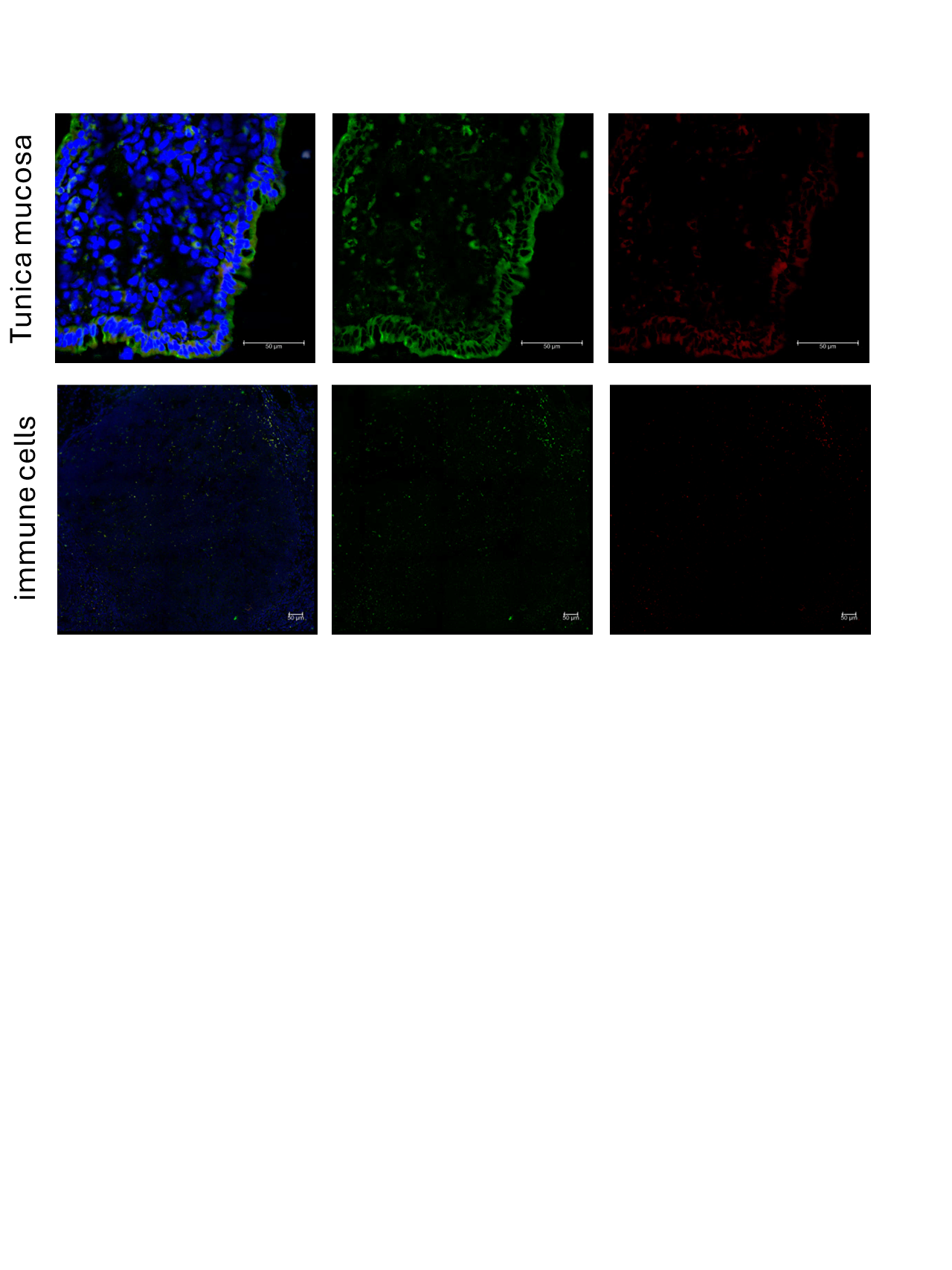

Supplement: S6 Fig — Frozen sections of the ileum were stained with FFAR3 (green) and FFAR2 (red) and a strong labelling of FFAR3 was found in the enterocytes of the tunica mucosa. bar [50μm]. (TIF) [file pone.0307411.s006.tif]

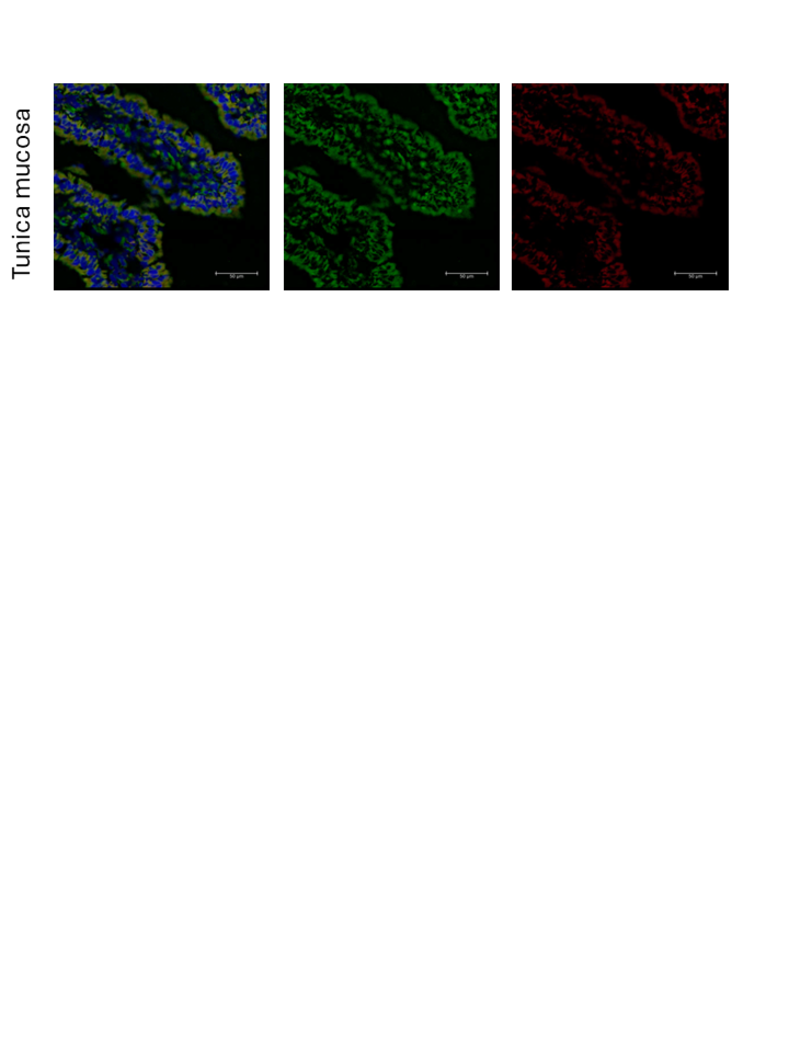

Supplement: S7 Fig — Frozen sections (animal 4) of the jejunum were labelled with antibodies for FFAR2 (red) and FFAR3 (green). Additionally, DAPI war used for nucleus staining. bar [= 50 μm]. (TIF) [file pone.0307411.s007.tif]

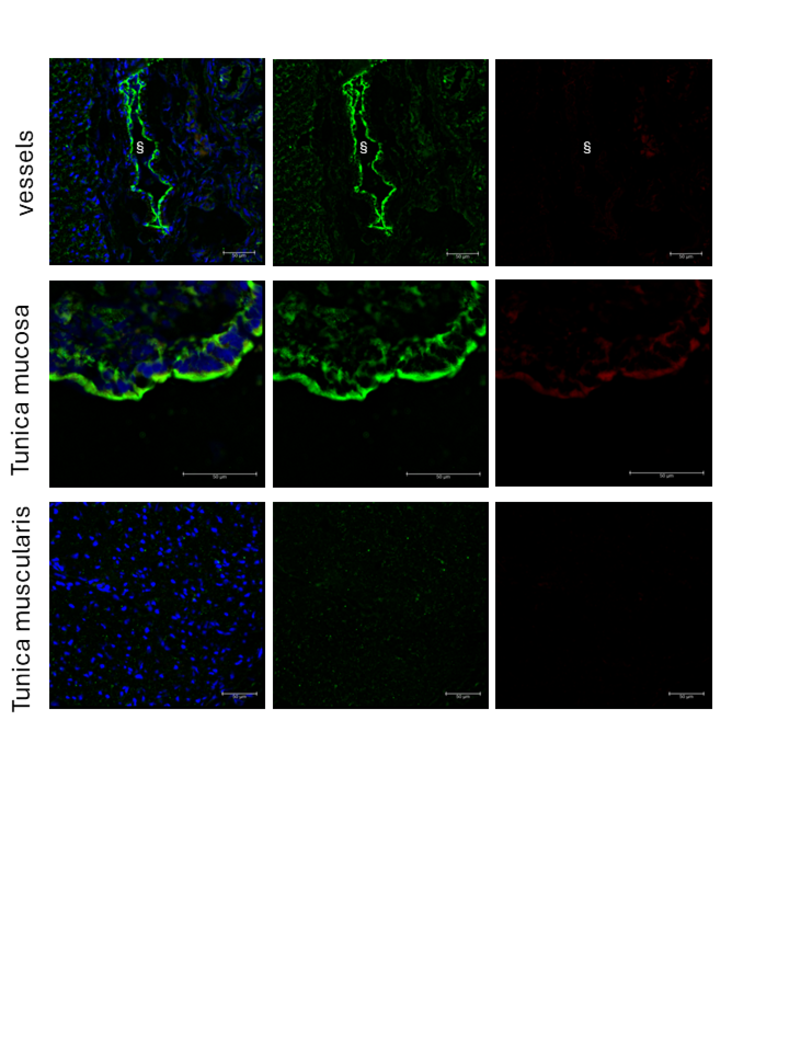

Supplement: S8 Fig — Using immunofluorescence labelling, distribution of FFAR2 (red) and FFAR3 (green) was made visible in frozen sections of ileum (animal 4). Furthermore, nuclei were stained with DAPI (blue). Endothelial cells of veins (§) showed a strong expression of FFAR3 (green) in contrast to FFAR2 (red). Additionally, enterocytes in the tunica mucosa expressed FFAR3 to a greater extent than FFAR2. Smooth muscle cells expressed only FFAR3 but not FFAR2. bar [= 50 μm]. (TIF) [file pone.0307411.s008.tif]

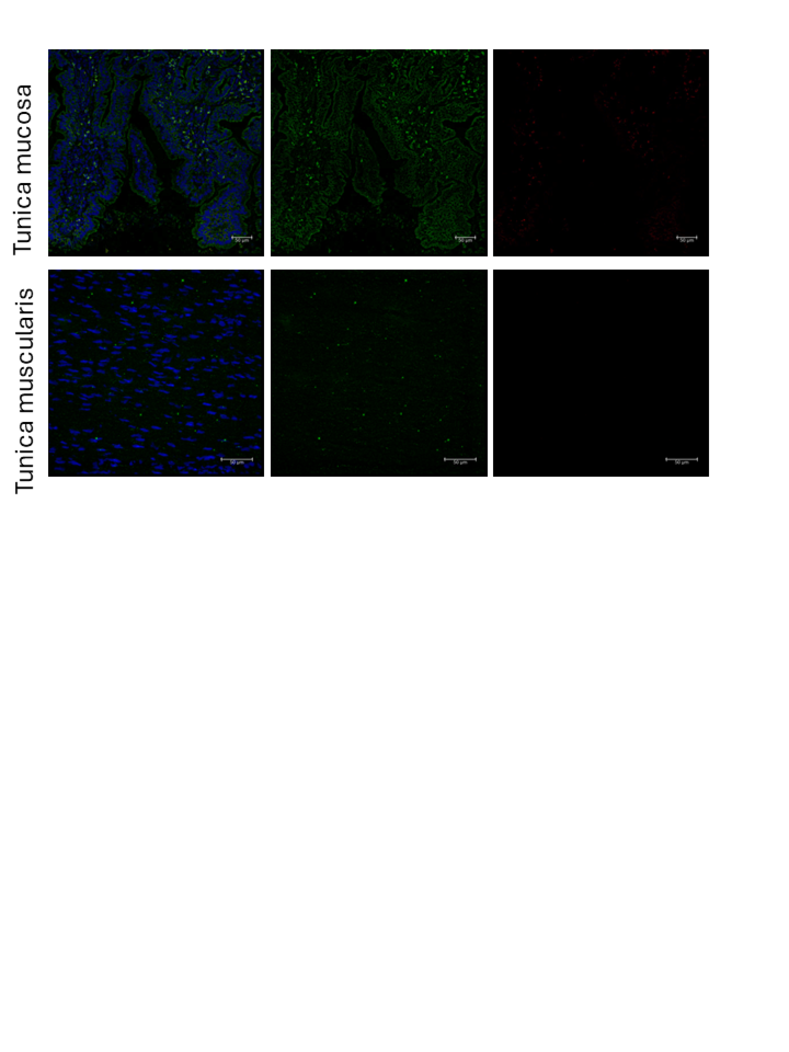

Supplement: S9 Fig — Additionally, DAPI war used for nucleus staining. bar [= 50 μm]. (TIF) [file pone.0307411.s009.tif]

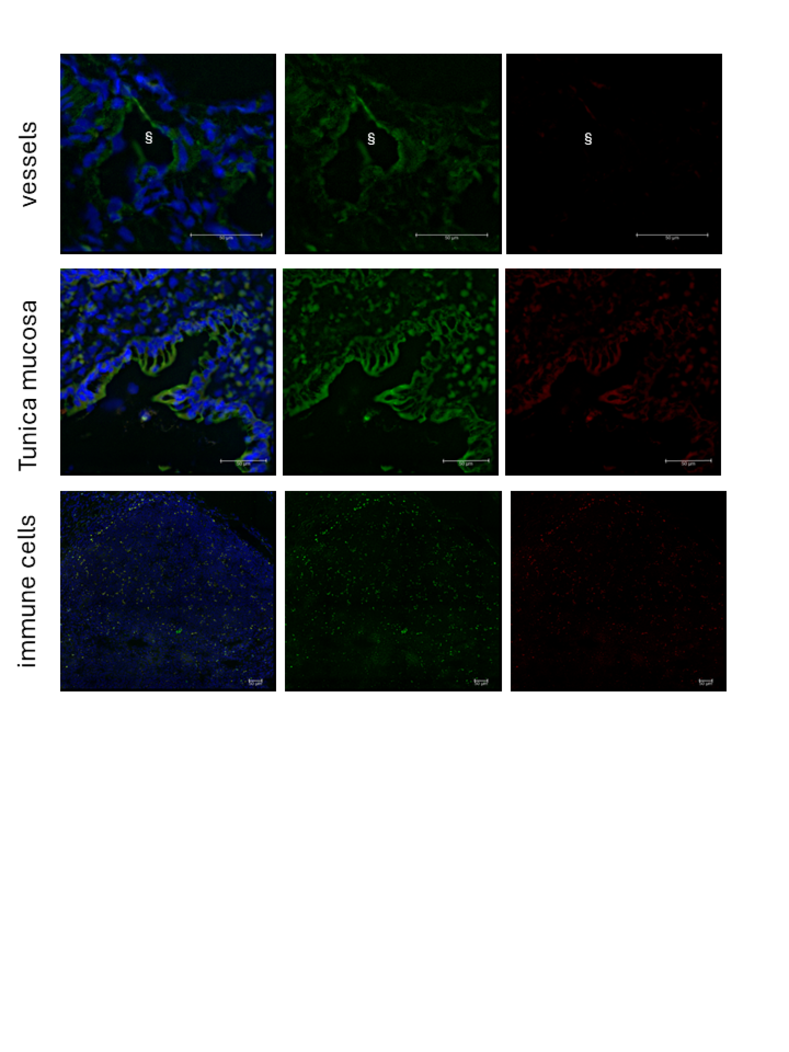

Supplement: S10 Fig — FFAR3 was strongly expressed in the tunica mucosa in contrast to FFAR2 (animal 5). Furthermore, a strong staining of endothelial cells was found in veins (§). The appearance of the cross sections of this vessel can lead to the conclusion that it could also be a lymphatic vessel but not a vein. (TIF) [file pone.0307411.s010.tif]

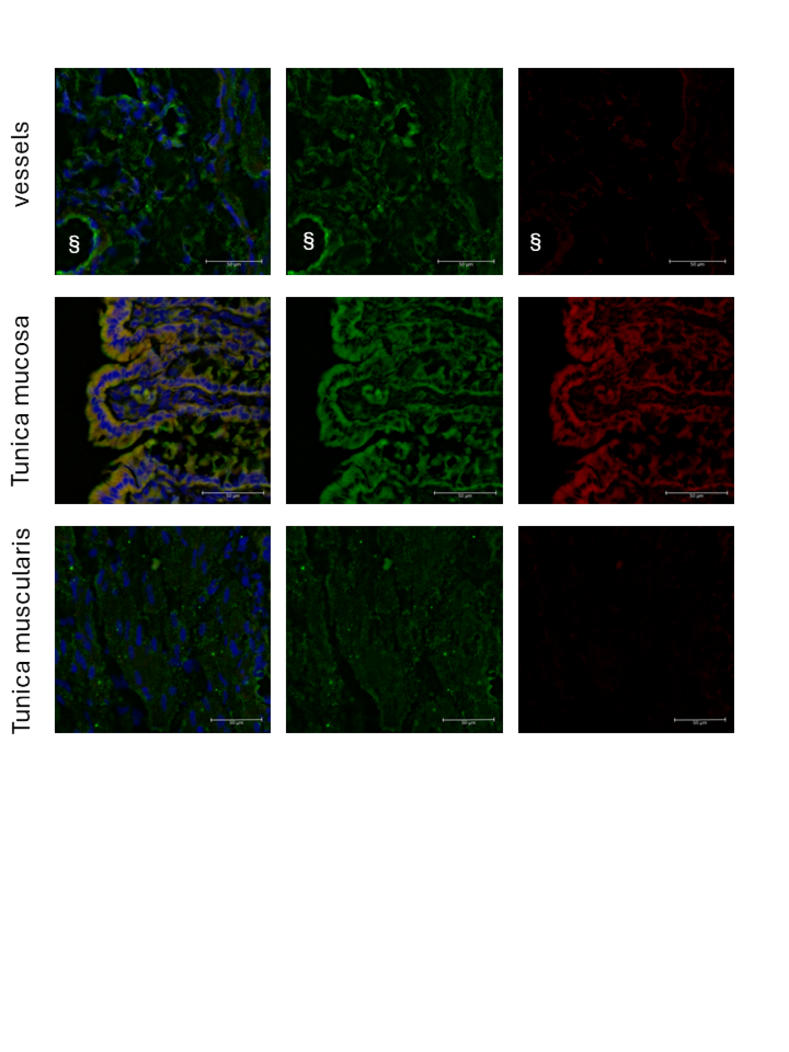

Supplement: S11 Fig — The tunica mucosa of the colon showed both: a strong FFAR3 and a strong FFAR3 expression (animal 5). The smooth muscle cells in the tunica muscularis showed no or only a weak expression of FFAR2. Endothelial cells of veins (§) were strongly labelled with FFAR3 but not with FFAR2. bar [50μm]. (TIF) [file pone.0307411.s011.tif]

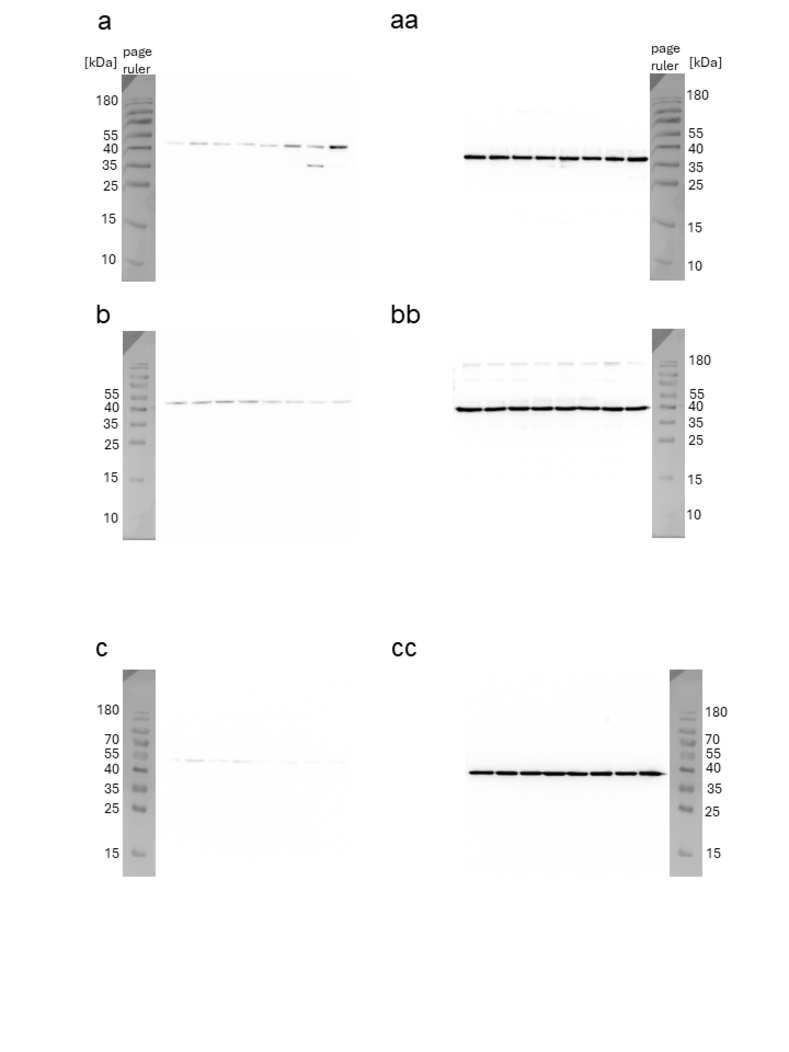

Supplement: S12 Fig — Western blot analyses were repeated (N = 3). Raw western blots of FFAR2 (predicted protein size 50 kDa, a-c) were shown with prestained page ruler. ß-actin (aa-cc) was used as loading control (38 kDa). (TIF) [file pone.0307411.s012.tif]

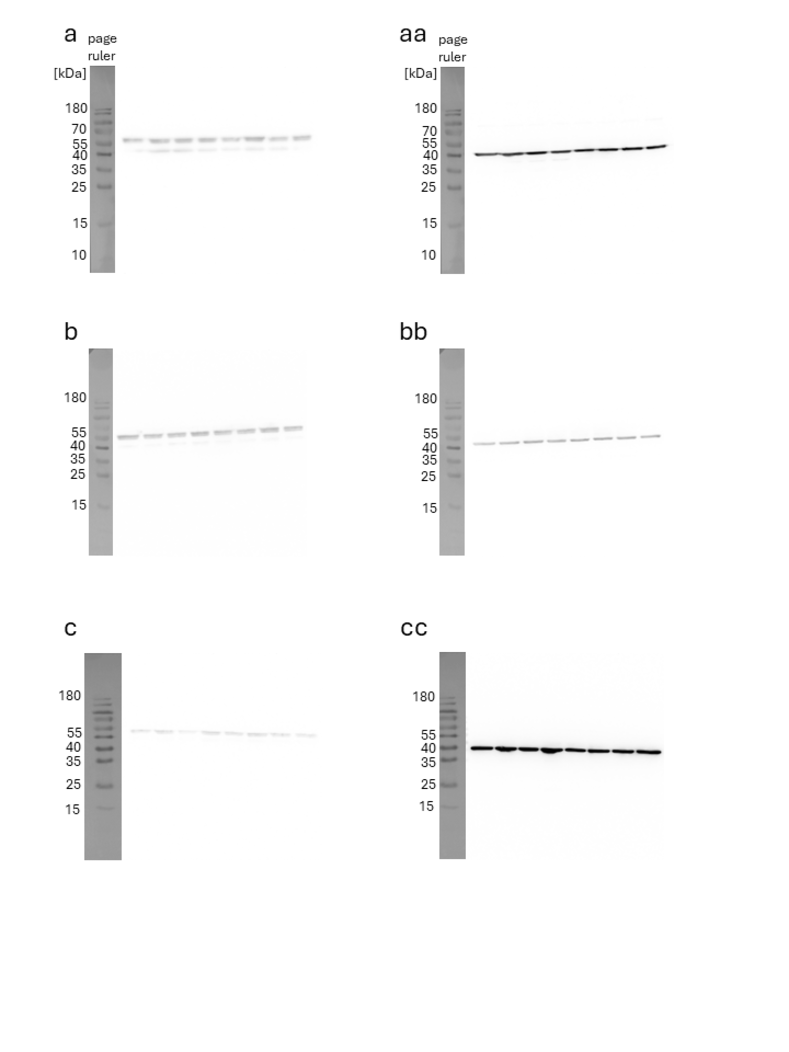

Supplement: S13 Fig — Western blot analyses were repeated (N = 3). Raw western blots of FFAR3 (a-c) were shown with prestained page ruler. ß-actin (aa-cc) was used as loading control (38 kDa). Protein samples of FFAR2 and FFAR3 were loaded on separated gels due to the similarity of the protein size. (TIF) [file pone.0307411.s013.tif]
