# Supplementary material for: Modeling of culture conditions by culture system, glucose and propionic acid and their impact on metabolic profile in IPEC-J2
Source: PLoS One. 2024 Jul 18;19(7):e0307411. doi: 10.1371/journal.pone.0307411 (PMC11257281; doi:10.1371/journal.pone.0307411)
Supplement: S1 Table — Main and interaction effects are shown in the table. The glucose content and cultivation as ALI or SMC showed significant effects on TEER values. The results based on 6 independent experiments. (DOCX) [file pone.0307411.s014.docx]

| **treatment** | **attributable variance** | **square sum** | **F** | **p-value** |
| --- | --- | --- | --- | --- |
| CON vs. ALI | 6.95% | 14.56 | 5.80 | 0.02 |
| HIGH vs. LOW | 40.22% | 84.32 | 33.59 | <0.001 |
| wo PA vs. PA | 0.35% | 0.73 | 0.29 | 0.59 |
| CON vs. ALI x HIGH vs. LOW | 3.09% | 6.47 | 2.58 | 0.12 |
| CON vs. ALI x wo PA vs. PA | 0.47% | 0.98 | 0.39 | 0.54 |
| HIGH vs. LOW x wo PA vs. PA | 0.93% | 1.95 | 0.78 | 0.38 |
| CON vs. ALI x HIGH vs. x wo PA vs. PA | 0.1% | 0.21 | 0.09 | 0.77 |
