# Supplementary material for: Modeling of culture conditions by culture system, glucose and propionic acid and their impact on metabolic profile in IPEC-J2
Source: PLoS One. 2024 Jul 18;19(7):e0307411. doi: 10.1371/journal.pone.0307411 (PMC11257281; doi:10.1371/journal.pone.0307411)
Supplement: S2 Table — A three-way ANOVA was performed to analyse the OCR of the baseline. A significant interaction effect was found between cultivation and glucose (N = 3). (DOCX) [file pone.0307411.s015.docx]

| treatment | attributable variance | square sum | F | p-value |
| --- | --- | --- | --- | --- |
| CON vs. ALI | 2.05% | 67567 | 0.656 | 0.427 |
| HIGH vs. LOW | 11.7% | 384779 | 3.74 | 0.068 |
| wo PA vs. PA | 3.73% | 122759 | 1.19 | 0.288 |
| CON vs. ALI x HIGH vs. LOW | 16.6% | 546801 | 5.31 | 0.032 |
| CON vs. ALI x wo PA vs. PA | 2.61% | 85756 | 0.833 | 0.372 |
| HIGH vs. LOW x wo PA vs. PA | 0.137% | 4494 | 0.0436 | 0.837 |
| CON vs. ALI x HIGH vs. x wo PA vs. PA | 1.92% | 63229 | 0.614 | 0.442 |
