# Supplementary material for: Modeling of culture conditions by culture system, glucose and propionic acid and their impact on metabolic profile in IPEC-J2
Source: PLoS One. 2024 Jul 18;19(7):e0307411. doi: 10.1371/journal.pone.0307411 (PMC11257281; doi:10.1371/journal.pone.0307411)
Supplement: S3 Table — A new ANOVA (two-way) was performed under consolidation of the propionate group. A significant main effect of the cultivation (CON vs. ALI) was observed but also a significant interaction effect between cultivation and glucose content. (DOCX) [file pone.0307411.s016.docx]

| treatment | attributable variance | square sum | F | p-value |
| --- | --- | --- | --- | --- |
| CON vs. ALI | 13.95% | 459303 | 4.719 | 0.040 |
| HIGH vs. LOW | 2.24% | 73869 | 0.7590 | 0.392 |
| CON vs. ALI x HIGH vs. LOW | 12.84% | 422719 | 4.343 | 0.048 |
