# Supplementary material for: Modeling of culture conditions by culture system, glucose and propionic acid and their impact on metabolic profile in IPEC-J2
Source: PLoS One. 2024 Jul 18;19(7):e0307411. doi: 10.1371/journal.pone.0307411 (PMC11257281; doi:10.1371/journal.pone.0307411)
Supplement: S4 Table — A three-way ANOVA was performed with the baseline values of the ECAR (N = 3). (DOCX) [file pone.0307411.s017.docx]

| treatment | attributable variance | square sum | F | p-value |
| --- | --- | --- | --- | --- |
| CON vs. ALI | 27.08% | 277132 | 9.12 | 0.007 |
| HIGH vs. LOW | 1.39% | 13904 | 0.458 | 0.506 |
| wo PA vs. PA | 2.26% | 22574 | 0.743 | 0.399 |
| CON vs. ALI x HIGH vs. LOW | 0.0398% | 398 | 0.0131 | 0.910 |
| CON vs. ALI x wo PA vs. PA | 0.885% | 8836 | 0.291 | 0.596 |
| HIGH vs. LOW x wo PA vs. PA | 3.76% | 37534 | 1.24 | 0.280 |
| CON vs. ALI x HIGH vs. x wo PA vs. PA | 0.222% | 2213 | 0.0729 | 0.790 |
